# Abstracts from the 6th International Conference for Healthcare and Medical Students (ICHAMS)

**DOI:** 10.1186/s12919-017-0071-z

**Published:** 2017-05-09

## O1 Assessing how a novel histone deacetylase 6 (HDAC 6) inhibitor kills chemoresistant cancer cells

### Sian Won Tan^1^, Candice Fraser^1^, Triona Ni Chonghaile^2^

#### ^1^School of Medicine, Royal College of Surgeons in Ireland, Dublin, Ireland; ^2^Department of Physiology and Medical Physics, Royal College of Surgeons in Ireland, Dublin, Ireland

##### **Correspondence:** Sian Won Tan

**Introduction:** Breast cancer is the most common cancer in women. A subtype of breast cancer called triple negative breast cancer (TNBC), has a poor response to treatment. Initially, the patient responds to chemotherapy but all too often the patients relapse and then become resistant. Dr. Ni Chonghaile identified a small molecule, BAS2 that could kill chemoresistant triple negative breast cancer cells but not kill non-transformed normal breast epithelial cells. The aim of this study was to understand how this small molecule kills breast cancer cells.

**Methods:** To assess how the HDAC6 inhibitor kills cells we used the TNBC cell line MDA-MB-231. MDA-MB-231 were either treated with increasing concentrations of the HDAC6 inhibitor, BAS2 alone or in combination with caspase inhibitors, QVD-OPH and z-VAD-FMK. Proteins were extracted, quantified, immunoblotted and then probed for acetylated alpha-Tubulin, total alpha-Tubulin, caspase-8 and PARP by Western blotting. The cell viability of the cells was assessed by flow cytometry with Annexin V and Propidium iodide (PI) staining.

**Results:** BAS2 caused an increase in the acetylation of alpha-tubulin. BAS-2 caused a dose-dependent increase in cell death. The cell death was inhibited by the treatment with caspase inhibitors (z-VAD-FMK and QVD-OPH). BAS2 in a dose-dependent manner caused cleavage of caspase 8 and PARP, and increase in cell viability when inhibited by caspase inhibitors.

**Discussion:** BAS-2 causes an increase in acetylation of alpha-tubulin indicating inhibition of HDAC6. Caspase inhibitor can protect against HDAC6 inhibitor induced-cell death. BAS2 could potentially kill cells through extrinsic apoptotic pathway as caspase-8 is cleaved following the BAS2 treatment.

## O2 The prognostic role of serum albumin in patients with advanced malignancy at referral to palliative care

### Barbara Giles^1,2^, Noreen O’Shea^2^, Tony O’Brien^2^

#### ^1^School of Medicine, University College Cork, Cork, Ireland; ^2^Palliative Medicine, Marymount University Hospital & Hospice, Cork, Ireland

##### **Correspondence:** Barbara Giles

**Introduction:** Patients with advanced malignancy are referred to Palliative Care for symptom management, psychological support and future care planning. Prognostication can be uncertain, however can be a driving force for decision-making regarding treatment and management. Serum albumin levels have been reported as one of the top five predictors of survival. This project aims to investigate prognostication using serum albumin levels in Palliative Care patients with malignancy.

**Methods:** A retrospective chart review was conducted at Marymount Hospice of inpatients who had died over a six-month period with malignancy. Data obtained included: patient demographics; referral date to Palliative Care; date of death; and serum albumin level at referral. Data was analysed using univariate and multivariate analyses in Excel and SPSS.

**Results:** 186 charts were included. The mean survival of patients was 24 days with serum albumin levels of <25g/L (95% CI 10 - 38 days), 81 days with 26-35g/L (95% CI 48 - 114 days), and 202 days above the normal level of 35g/L (95% CI 157 - 246 days). Serum albumin levels are directly linked to the number of days spent in palliative care (p <0.0001), with every unit increase in albumin correlating to an increase in survival of 11.2 days. Age is also a prognostic factor, as each increase in year correlates to a decrease of 2.9 days in survival.

**Discussion:** Serum albumin levels are a statistically significant prognostic indicator in the Palliative Care population with advanced malignancy, and thus provide an approximated time frame of survival for physicians, patients and caregiver at referral to Palliative Care.

## O3 Does age influence the duration of mechanical ventilation post coronary artery bypass surgery?

### Donnacha Hogan^1^, Shane O’Driscoll^2^, Paul Cullen^2^, Kishore Doddakula^2^

#### ^1^Medical School, University College Cork, Cork, Ireland; ^2^Cardiothoracic Department, Cork University Hospital, Cork, Ireland

##### **Correspondence:** Donnacha Hogan

**Introduction:** Age is known to be a major risk factor for patients undergoing cardiac surgery, however its precise risks are incompletely understood. The aim of the present study was to assess the impact of age on the duration of mechanical ventilation following Coronary Artery Bypass Grafting (CABG).

**Methods:** A retrospective cohort study was performed. 2 groups (Group-O ≥ 70-years-old, Group-U < 70-years-old) were created and the most recent 100 patients in each group undergoing isolated CABG up to 30/06/16 were included. Patients undergoing emergency surgery or who had previous cardiac surgery were excluded. Following ethical approval, data were obtained from computerised databases (ICIP,iPIMS,PATS). Statistical analysis was performed using SPSS-v22.

**Results:** Group-O demonstrated a significant increase in ventilation time (14.364 ± 5.927 hours vs 11.418 ± 5.459 hours, p = 0.001), with a correlating increase in ICU length of stay (43.55 [23-88.56] hours vs 23.55 [20.5-43.92] hours, p = <0.001) and the time to be discharged home (9 [7-13] days vs 7 [6-10] days, p = <0.001). However there was not a significant difference in the total length of stay in hospital (14 [10-24] days vs 13 [8-22] days, p = 0.089). Notably 3.7% (p = 0.006) of the variance in ventilation time can be explained by one’s age. Each one year increase in age was associated with an 8.28 minute increase in ventilation time.

**Discussion:** Age appears to impact the duration of mechanical ventilation, ICU length of stay and time to discharge in patients aged 70-year-of-age and older. Total length of stay in hospital is not affected, perhaps indicating surgery is expedited for older patients once they are in hospital. It is important that these data be acknowledged in order to allow adequate time to be allocated for the recovery of older patients. This information is particularly salient given the current strain on resources and scarcity of ICU beds.

## O4 Hormonal Therapy Medication Taking Behaviour (MTB) and side-effects in women with Stage I to III Breast Cancer in Ireland

### Nicole Wan June Fong^1^, Kathleen Bennett^2^, Stephan Dombrowski^3^, Linda Sharp^4^, Caitriona Cahir^2^

#### ^1^School of Medicine, Royal College of Surgeons in Ireland, Dublin, Ireland; ^2^Division of Population Health Sciences, Royal College of Surgeons in Ireland, Dublin, Ireland; ^3^Division of Psychology, University of Stirling, Stirling, UK; ^4^Institute of Health and Society, Newcastle University, Newcastle upon Tyne, UK

##### **Correspondence:** Nicole Wan June Fong

**Introduction:** Women with estrogen positive breast cancer are recommended to undergo 5-10 years of adjuvant hormonal therapy to prevent cancer recurrence and mortality. Despite the proven clinical efficacy of hormonal therapy, many women do not take their treatment as recommended. The aim of this study was to investigate the association between hormonal therapy medication taking behaviour (MTB) and side-effects in women with stage I to III breast cancer.

**Methods:** A cross-sectional national population-based study of women with stage I-III breast cancer prescribed hormonal therapy in Ireland was conducted. Participants were identified from the National Cancer Registry Ireland database and invited to complete a postal questionnaire measuring; (i) demographics; (ii) self-reported MTB; and (iii) endocrine side-effects (FACT-ES). The Irish Congress of General Practitioners granted ethical approval. The association between MTB and side-effects was assessed using Poisson regression analysis with adjustment for demographic and clinical covariates.

**Results:** In total 1,612 women completed the questionnaire (response rate = 65%); 1,207 (74.9%) women were adherent and persistent; 178 (11%) women were non-adherent but persistent and; 227 (14.1%) women were non-persistent. The median number of side-effects was 5 (IQR: 2, 8). There was significantly increased risk in the expected number of side-effects for women who were non-adherent but persistent with their HT, after adjusting for covariates (adjusted IRR 1.16; 95% CI 1.05, 1.29, p < 0.01).

**Discussion:** Healthcare professionals need to ease the burden of hormonal therapy side-effects in order to improve MTB and health outcomes in women with breast cancer.

## O5 Developing a sustainable deprescribing program at the Michael Garron Hospital

### Mark Daubaras^1^, John Abrahamson^2^

#### ^1^School of Medicine, Royal College of Surgeons of Ireland, Dublin, Ireland; ^2^Hospitalist Medicine, Michael Garron Hospital, Toronto, Canada

##### **Correspondence:** Mark Daubaras

**Introduction:** Numerous studies have shown that, in elderly patients, certain classes of medications may have reduced therapeutic benefit or harmful side effects. Deprescribing is the systematic discontinuing of drugs where their potential for harm outweighs their potential benefits. This study aimed to develop a business model to demonstrate the feasibility of developing a sustainable deprescribing program at the Michael Garron Hospital (MGH).

**Methods:** All admissions to the General Medicine Service at the MGH over a 30 day period were enrolled with a review of home prescriptions conducted on participants over the age of 65 and prescribed more than five medications. The total numbers of recommendations were recorded; estimated savings were calculated. Readmissions within 30 days were reviewed to determine if this was a consequence of the deprescibing recommendations. Compliance to suggested medication changes was tracked through the Ontario Drug Database.

**Results:** From 272 admissions, 72 participants (26%) underwent full review of their home prescriptions with 255 total deprescribing recommendations made. Among the reviewed participants, less than one in ten readmissions within 30 days was attributed to a deprescribing recommendation. Estimated annual savings to the hospital as a result of this deprescribing pilot was $18,000CAD.

**Discussion:** Approximately 25% of Hospitalists patients at MGH could benefit from a deprescribing review. We have also verified that an accepting culture for a deprescribing program. Readmissions within 30 days were rarely linked to a deprescribing recommendation. Our pilot demonstrates that a business model for hospitals to develop a sustainable deprescribing program can be funded through pharmaceutical cost savings.

## O6 Pre-clinical Investigation of the Effects of a Novel Compound on the Expression of CD68 and CD163 in Experimental Autoimmune Vasculitis

### Jiaying Lau, Mark Little

#### Trinity Health Kidney Centre, Trinity College Dublin, Dublin, Ireland

##### **Correspondence:** Jiaying Lau

**Introduction:** Anti-Neutrophil Cytoplasmic Autoantibody (ANCA) associated vasculitis is a rare autoimmune condition primarily affecting the kidney. ANCA vasculitis affects 1 in 50,000 people with a 24% 5-year mortality rate, highlighting the need for new therapies. This research aims to investigate the effect of a novel compound (Compound X) on CD68 (lysosomal/endosomal marker) and CD163 (monocyte/macrophage marker, typically M2 macrophages) in Experimental Autoimmune Vasculitis (EAV), a rat model used in ANCA vasculitis. We hypothesized that 10mg/kg/week Compound X would be able to ameliorate the inflammation process.

**Methods:** Wistar-Kyoto (WKY) rats (n = 13) were immunised with 3.2mg/kg of human Myeloperoxidase (MPO) to induce EAV. Rat kidney and spleen sections (8μm) were fixated with Periodate Lysine Paraformaldehyde. Healthy controls were compared against EAV models treated with 4 weeks of either vehicle or 10mg/kg/week compound X. An indirect immunofluorescent assay was used to localise the CD68 and CD163 proteins. Cells staining positive for CD68, CD163 or both markers were counted blind in 30 consecutive glomeruli.

**Results:** EAV models treated with vehicle displayed 5-fold increase in double positive cells/glomerular cross section (GCS) compared to healthy controls (mean = 0.0878 vs 0.013; P = 0.02). EAV treated with Compound X shows no significant differences in double positive cells compared to healthy controls (P = 0.76). CD68 expression statistically persisted after Compound X treatment (P = 0.31).

**Discussion:** Anti-inflammatory M2 macrophages were significantly increased in full-blown EAV, indicating current inflammation. After treatment with Compound X, the inflammation has been resolved indicated by decreasing double positive cells. Persisting CD68 expressions point towards non-myeloid cell types involved in wound-healing (e.g. fibroblasts).

## O7 The development of anti-Staphylococcal compounds for the treatment of Methicillin Resistant Staphylococcus aureus (MRSA)

### Niamh Kyne, Afnan Ali, Marian Brennan

#### Molecular and Cellular Therapeutics, Royal College of Surgeons in Ireland, Dublin, Ireland

##### **Correspondence:** Niamh Kyne

**Introduction:** The platelet receptors FcγRIIa and GPIIb/IIIa, play vital roles in platelet activation and contribute to S.aureus-mediated sepsis and thrombocytopenia. ClfB, a surface protein on S.aureus facilitates colonisation of the bacterium by binding to exposed fibrinogen in wounds and human cytokeratin 10 in the anterior nares. ClfB triggers platelet activation by binding to GPIIb/IIIa receptors on platelets via fibrinogen cross-bridges. S.aureus can also activate platelets by binding to FcγRIIa using IgG linking. This interaction is regarded as key to the development of sepsis. It is also involved in the pathogenicity of autoimmune diseases such as systemic lupus erythematosus and rheumatoid arthritis.

**Methods:** In this project, a novel drug (MB05) that blocks binding of ClfB to fibrinogen was analysed in platelet aggregations and using Isothermal Titration Calorimetry (ITC). The research also involved screening of approved drugs with potential to inhibit the FcγRIIa receptor and thus, IgG-mediated activation of platelets. Screening was conducted using IgG adhesion assays and platelet aggregations.

**Results:** The approved drug caspofungin fully inhibited platelet-IgG binding in adhesion assays at 33uM. A log-dose response curve was generated for the drug and the IC50 was established, 3.6uM, which fell within the therapeutic range for caspofungin. Roxithromycin and erythromycin drugs were potent inhibitors of platelet aggregation using S.aureus as agonist.

**Discussion:** The ability of Caspofungin to inhibit platelet adhesion to IgG, demonstrated an inhibitory effect on the FcγRIIa receptor. The inhibitory effects of roxithromycin and erythromycin suggest that these antibiotics could be used in sepsis not only as antibacterial agents, but also to improve haemostasis.

## O8 A preliminary study of left ventricular dysfunction in a cohort of UK Marfan Syndrome patients

### Carlos Gracias^1^, Oswalso Valencia^2^, Jose Aragon-Martin^3^, Yukiko Isekame^3^, Alex Wan^3^, Anne Child^3^

#### ^1^School of Medicine, Royal College of Surgeons in Ireland, Dublin, Ireland; ^2^Medicine, St. George’s University Hospital, London, UK; ^3^Marfan Trust UK, London, UK

##### **Correspondence:** Carlos Gracias

**Introduction:** Marfan syndrome is an inherited connective tissue disorder, caused by mutations in the FBN-1 gene, which manifests in several organ systems including the cardiovascular system. However, there is conflicting evidence about whether the disease directly alters left ventricular function and size. The aim of this study was to conduct a preliminary investigation as to whether Marfan Syndrome causes a primary abnormality in left ventricular function and dimensions.

**Methods:** 37 Marfan Syndrome patients and 43 age and sex-matched healthy controls were retrospectively studied using conventional echocardiography. Various variables were analysed to investigate possible differences in left ventricular systolic and diastolic function, as well as changes in left ventricular size.

**Results:** No statistically significant differences were found between the two groups, with the exception of an increased diameter of the Sinus of Valsalva in the Marfan Syndrome patients.

**Discussion:** Although there were no statistically significant differences in LV function and size between the Marfan Syndrome and control groups (except in the diameter of the Sinus of Valsalva), further study has been indicated by the results. In future investigations, limitations of the present study must be curbed, and a larger population of FBN-1 mutation proven individuals should be analysed with novel echocardiography techniques such as speckle-tracking echocardiography.

## O9 The Epidemiological Features of Herpes Simplex Virus Cases in a Cork STI Clinic

### Jill Mitchell^1^, Suzanne Cremin^2^

#### ^1^School of Medicine, University College Cork, Cork, Ireland; ^2^G.U.M/S.T.D. Clinic, South Infirmary-Victoria University Hospital, Cork, Ireland

##### **Correspondence:** Jill Mitchell

**Introduction:** Herpes simplex virus (HSV) is the leading cause of genital lesions worldwide. The transmission of sexually transmitted infections (STIs) and human behaviour are intrinsically linked. Therefore, a clear understanding of the behavioural and demographic characteristics that increase the risk of acquiring these infections is vital for effective STI control.

This study aimed to establish risk factors for the acquisition of genital HSV infection in Ireland.

**Methods:** A retrospective chart review, examining demographic, behavioural and diagnostic data of patients who attended a Cork STI clinic from 2011 to 2015 inclusive. Multivariate logistic regression models were used to study the epidemiological features of patients with a genital HSV infection (N = 296) in comparison to a control population of patients with negative screen (N = 307).

**Results:** Females (OR: 3.942, P < 0.001) and those aged between 25 to 30 years (OR: 8.397, P < 0.001) had increased risk of acquiring genital HSV. Subjects of non-Irish ethnicity (P = 0.032) and females who engaged in sexual intercourse before 17 years of age more likely to present with genital HSV (OR: 7.427, P < 0.01). High number of sexual partners was not found to increase the risk of genital HSV infection. Classic risk taking behaviours were not significant predicting factors of HSV infection. Consistent condom use was very low in all subjects.

**Discussion:** Public health campaigns directed at young women, especially those engaging in sexual activity at a young age may be beneficial. Campaigns should ensure to reach non-Irish ethnic groups. Increased distribution of condoms to at risk age groups should be considered.

## O10 Bioreactor-aided Pre-conditioning of Human Mesenchymal Stem Cells for Cartilage Regeneration: A pilot study to optimise the culture protocol

### Lavanya Venkatesh^1^, Wade Suen^2^, Bin Wang^2^, Sien Lin^2^, Wayne YW Lee^2^

#### ^1^School of Medicine, Royal College of Surgeons, Dublin, Ireland; ^2^Department of Orthopaedics and Traumatology, Prince of Wales Hospital, Chinese University Hong Kong, Hong Kong, Hong Kong

##### **Correspondence:** Lavanya Venkatesh

**Introduction:** The two fundamental research questions in mesenchymal stem cells (MSCs) based cartilage regeneration are how to simplify MSCs expansion and enhance chondrogenic potential. We have developed a novel chondrogenic pre-conditioning method to enhance MSC’s chondrogenic ability. This pilot study aims to examine the feasibility to conduct chondrogenic pre-conditioning in bioreactor culture to provide a simplified solution to obtain sufficient number of chondrogenic MSCs for tissue engineering.

**Methods:** MSCs were encapsulated by mixing with 2% w/v alginate. 1mL alginate containing 4x104 cells was added into chitosan by a needle in a drop wise manner. The same portion of encapsulated MSCs were cultured in a bioreactor containing a chondrogenic medium and in conventional culture plates as control for 7 days. Cytotoxicity, cell content and chondrogenic potential was assessed by live/dead cells staining, DNA measurement and qPCR, respectively. One-way ANOVA with Tukey post-hoc analysis was conducted.

**Results:** Bioreactor culture enhanced cell proliferation without causing significant cytotoxicity (N = 3). Chondrogenic ability of the cells were also assessed by testing for transcription factor Sox9, and extracellular components type II collagen and aggrecan, where the bioreactor culture showed a 2-fold improvement compared to the control (N = 5).

**Discussion:** This preliminary study showed that bioreactor culture of encapsulated MSCs was not cytotoxic and could enhance MSCs proliferation. The encapsulated MSCs were also responsive to chondrogenic induction. Further study is warranted to optimise the seeding density of MSCs in the microsphere and to evaluate the chondrogenic ability after prolonged culture.

## O11 Futile medication use in Palliative patients at the time of hospice referral

### Sarah Mabelson^1^, Tony O’Brien^2^

#### ^1^Medicine, University College Cork, Cork, Ireland; ^2^Palliative Medicine, University College Cork, Cork, Ireland

##### **Correspondence:** Sarah Mabelson

**Introduction:** When patients transition to palliative services the aims of care shift and drug indications may change, putting patients at risk of therapeutic futility. This study evaluated therapeutic futility in palliative patients at time of referral to Marymount Hospice.

**Methods:** This study was a retrospective chart review of consecutive adult inpatients who died in the hospice over a 6-month period. The pre-admission medication profiles were collected from charts, along with relevant cohort descriptors. A modified version of the Medication Appropriateness Index was applied to each medication to quantify its appropriateness. The pre-admission drug profile was then cross referenced with the inpatient kardex to record suspended medications.

**Results:** Fifty-three patients met the inclusion criteria. A total of 514 medications were recorded across 44 classes. The mean number of medications per patient was 10.4 (range 2-16) with 48 (91%) patients taking 5 or more drugs. The MAI rated 120 (23%) medications as inappropriate, with 42 (79%) patients taking at least one inappropriate medication. Supplements were found to be significantly more inappropriate than other common drug classes. The most common reasons for inappropriateness were unacceptable duration of therapy (12%) and lack of indication (10%). A significant relationship was found between inappropriateness and drug suspension (p < 0.05).

**Discussion:** Polypharmacy is almost universal in this population, and therapeutic futility is common. Significant differences exist between the appropriateness of commonly prescribed drug classes. Some effort is being made to review and suspend inappropriate medications, however further research is needed to evaluate the potential role of prescription review guidelines for palliative care.

## O12 Barriers of proper mental health care delivery in Southeastern European Oncology Units

### Mariana Petrescu^1^, Alejandra Escoto^2^, Alexandra Sidor^3^

#### ^1^General Medicine, University of Medicine and Pharmacy Iuliu Hatieganu, Cluj-Napoca, Romania; ^2^College of Public Health, University of Iowa, Iowa City, IA, USA; ^3^Department of Public Health, Babes-Bolyai University, Cluj-Napoca, Romania

##### **Correspondence:** Mariana Petrescu

**Introduction:** Cancer patients’ health outcomes are negatively affected by their high levels of psychological distress. Factors related to health-care systems, to patients and to health practitioners represent important barriers in addressing these patients’ mental health needs. This study aims to investigate barriers - as perceived by health practitioners - in referring cancer patients from Southeastern European oncology clinics to mental health care.

**Methods:** This study was implemented in four state-owned cancer clinics from Albania, Moldova, Romania and Serbia. 24 medical oncologists and 8 psychotherapists and psychiatrists were interviewed. Three researchers with backgrounds in psychology, medicine and public health used an inductive approach and thematic analysis to code data.

**Results:** Participants perceived lack of healthcare regulations, poor knowledge towards these norms and their poor enforcement as main barriers in addressing cancer patients’ distress. The systematic problems our participants considered most concerning were the lack of human resources, of organization and of appropriate infrastructure for delivering mental health care within oncology units. Oncologists’ burnout and low mental health literacy were reported to pose barriers in screening patients for distress. Poor physician-patient communication and poor oncologist-psychologist communication were identified as further barriers related to health practitioners.

**Discussion:** Results prove the need for better strategies in providing mental health services for cancer patients in Southeastern Europe and indicate that for improving the quality of these services, barriers at all levels need to be addressed. The present results provide guidelines for future strategies to improve mental healthcare within oncology settings from Southeastern Europe.

## P1 Drug administration to a cohort of neonates <32 weeks gestation and cumulative exposure to potentially harmful excipients

### Fiona Aherne, Eugene Dempsey

#### Department of Paediatrics and Child Health, University College Cork, Cork, Ireland

##### **Correspondence:** Fiona Aherne

**Introduction:** Excipients in medications administered to neonates have been mostly evaluated for safety in adults, leading to potential adverse effects. Pre-term infants are especially vulnerable to these effects.

The aims of the project were to establish what drugs were prescribed to a cohort of neonates <32 weeks gestation in CUMH over five months; and to measure their exposure to harmful excipients.

**Methods:** A retrospective chart review was conducted on neonates <32 weeks born between January and May 2016. Data was extracted from drug kardexes, and the most commonly prescribed drugs were identified. Information regarding excipients of the relevant formulations was obtained from the SPCs. Excipients of interest were categorised from a literature review. These excipients were detected in the common drugs prescribed in the unit. The neonates that received these excipients were identified and quantitative information regarding their exposure was calculated.

**Results:** A total of 40 neonates were included in the study. Mean gestation was 28 weeks and birth weight 1143g. Antibiotics, caffeine and diuretics were identified as the most commonly prescribed drug groups. 52 drugs were prescribed during. 6 harmful excipients (e.g. ethanol) and 5 potentially harmful excipients (e.g. sodium metabisulphite) were identified. These were in 8 medications e.g. caffeine citrate oral solution contains ethanol 0.1%(w/v%) and was administered to 90% of neonates over a median duration of exposure of 40 days.

**Discussion:** The research yielded novel results regarding prescribing trends in the NICU, CUMH. Most formulations were free of excipients or contained excipients that are not known to be harmful. Some oral formulations contained harmful excipients that were widely administered.

## P2 An Audit of Ovulation Induction (OII) Programme in Cork University Maternity Hospital (CUMH) from 2015-2016

### Alya Ameera Azizul^1^, Moya McMenamin^2^

#### ^1^University College Cork, Cork, Ireland; ^2^Obstetrics & Gynaecology, Cork University Maternity Hospital (CUMH), Cork, Ireland

##### **Correspondence:** Alya Ameera Azizul

**Introduction:** Ovulation induction (OII) is a common first-line infertility treatment for women with ovulatory dysfunction. This research aims to review the overall effectiveness of OII programme in Cork University Maternity Hospital (CUMH) from 2015-2016. Objectives include (1) to identify ovulation induction agents used with evidence of effectiveness and complications, and (2) to assess possible contributing factors to successful conception.

**Methods:** A 2 year retrospective study (2015-2016) involving 44 patients referred for OII from the public fertility clinic in CUMH. Data on baseline fertility investigations, OII agents used including the dosage, side effects and outcomes of treatment cycles were obtained from patients’ medical records. Data was analyzed with SPSS.

**Results:** A total of 154 OII cycles in 44 patients were studied (127 with clomiphene citrate, 23 with FSH and 4 with Letrozole). Of the 44 patients, 40.9% (n = 18) achieved pregnancy. 44.4% of pregnancies (n = 8) resulted spontaneously and of those conceived via OII (n = 10), 60% (n = 6) resulted in singleton pregnancy and 10% (n = 1) multiple pregnancy. Unifollicular development was seen in 63.7% (n = 81) of clomiphene citrate cycles, 56.5% (n = 13) of FSH cycles and 100% (n = 4) of Letrozole cycles. Complication rate was highest with clomiphene citrate; endometrial thinning <0.6cm in 23.5% (n = 35), multiple follicles production in 13% (n = 20), ovarian cyst formation in 7.7% (n = 4) and OHSS in 3.8% (n = 2).

**Discussion:** OII is an effective fertility treatment for those with ovulatory dysfunction as the cause for subfertility with 40.9% achieving pregnancy. Clomiphene citrate remains the most commonly used OII agent and it shows the highest efficacy towards successful conception.

## P3 Audit of discharge prescriptions and sources of medication error on transition from an urban secondary care facility to primary care

### Eimear Connolly^1^, Elaine Walsh^2^

#### ^1^Medicine, University College Cork, Cork, Ireland; ^2^General Practice, University College Cork, Cork, Ireland

##### **Correspondence:** Eimear Connolly

**Introduction:** Medication errors are a recognised cause of preventable morbidity and mortality. Errors often occur as patients transition between hospital and primary care, and are particularly common in elderly patients, taking multiple medications. Accurately transferring information on discharge is challenging. We sought to audit the current model of discharge to identify the incidence and type of prescribing error occurring at discharge to the community, and suggest possible solutions.

**Methods:** Patients over the age of 65, with minimum of 3 admission medications, and who attended the investigating secondary care unit were included. Once recruited, the patients’ existing medications were verified with their GP and community pharmacy. On discharge, a retrospective chart review was carried out to collect demographic data, and examine the inpatient drug record, discharge prescription and discharge letter, recording any discrepancies or errors.

**Results:** 64 patients were recruited, 38 male and 28 female. 3 patients were excluded due to incomplete discharge information. The mean age of participants was 77.34 years (SD 7.36 years). The mean number of medications on admission was 10.53, SD 5.02, range 3-24. The number of discharge medications was lower, with a mean of 8.02, SD 4.25 and range 1-21. Including items omitted erroneously, error of any kind was recorded on 96% of written discharge prescriptions.

**Discussion:** Prescribing error is almost ubiquitous on handwritten discharges, with omitted items, lack of prescriber information, and lack of treatment dosage and duration the most commonly encountered errors. Reform of discharge prescribing procedures should be targeted to improve prescribing accuracy and patient safety.

## P4 Incidence of Gluteus Medius and Minimus Tendinosis on MRI: Consideration for Needle Fenestration

### Brendon Ginzburg^1^, Christine Macadam^2^, Lawrence Friedman^2^

#### ^1^School of Medicine, Royal College of Surgeons in Ireland, Dublin, Ireland; ^2^Diagnostic Imaging, North York General Hospital, Toronto, Canada

##### **Correspondence:** Brendon Ginzburg

**Introduction:** Chronic pain about the greater trochanter can be severe and disabling. Tendinosis of the gluteus medius and minimus tendons is believed to be a major cause for this pain. Multiple treatments have been tried with variable success. A recent study showed gluteal tendon needle fenestration under ultrasound guidance improved symptoms in 82% of investigated patients. Fenestration performed on a limited number of patients by one of the authors of this current study showed encouraging early results. The purpose of this study was to determine the incidence of gluteal tendinosis in our local patient referral population.

**Methods:** A retrospective review of 50 hip MRI cases was performed by 2 experienced radiologists looking specifically for the presence or absence of gluteus medius and minimus tendinosis.

**Results:** Tendinosis was found in 68% of the patients. There was a female predominance with 78% of women and 43% of men showing tendinosis. The incidence of tendinosis increased with age, with no cases of tendinosis under age 36 and with the greatest percentage of tendinosis in patients between 51 and 70.

**Discussion:** Gluteal tendinosis was a common finding on MRI of the hips. Therefore, MRI finding of tendinosis alone would not be sufficient for referral for fenestration. Appropriate selection of patients for fenestration would require careful correlation between clinical presentation (location and quality of pain) and MRI findings.

## P5 A review of reproductive pregnancy outcomes of women with two consecutive miscarriages and no living child

### Deirdre Green^1^, Keelin O’Donoghue^2,3^

#### ^1^Medical School, University College Cork, Cork, Ireland; ^2^Pregnancy Loss Research Group, Department of Obstetrics and Gynaecology, Cork University Maternity Hospital, Cork, Ireland; ^3^The Irish Centre for Fetal and Neonatal Translational Research (INFANT), University College Cork, Cork, Ireland

##### **Correspondence:** Deirdre Green

**Introduction:** Recurrent miscarriage is usually defined as three consecutive first-trimester pregnancy losses. However, there is an increasing tendency to define those with two consecutive first-trimester miscarriages and no living child as also recurrent and in need of investigation. For expectant parents, any pregnancy loss is devastating and many request investigation irrespective of clinical guidance.

This study aimed to examine the management and subsequent pregnancy outcomes of nulliparous women who attended our Pregnancy Loss Clinic between 2009 and 2014, with their second consecutive first-trimester miscarriage.

**Methods:** A search of the Pregnancy Loss Clinic database identified women suitable for inclusion. Information was sourced from the database, the electronic hospital patient management and laboratory systems, and clinical letters. Risk factors identified during clinic visits, results of medical investigations, details of therapies initiated and subsequent pregnancy outcomes were recorded.

**Results:** We identified 294 women, with a mean age of 33.4 years (range 16 - 46). Following investigation, 69.4% had no identifiable cause found for their miscarriages. However, 56.8% of women were prescribed a pharmacological treatment. A subsequent pregnancy was achieved in 82.3% (242/294), with increased age and smoking status found to significantly affect the likelihood of conception (p = 0.017, p = 0.012 respectively). Of the 242 subsequent pregnancies, 72.7% (176/242) resulted in a live birth. Increased maternal age (p = 0.045) was found to significantly affect next pregnancy outcome.

**Discussion:** Current investigation is unlikely to identify an underlying pathology. The role of medical therapy in this cohort is not fully understood. Subsequent pregnancy outcomes are excellent.

## P6 Establishment of the expression of Green Flourescent Protein (GFP) & Luciferase in rat macrophages with a Lentiviral vector transduction

### James Griffin^1^, Michael Smith^2^, Qing Ma^2^, Zhiquan Zhang^2^, Janet Staats^3^, Brian Barnes^4^, Kent Weinhold^3^, Mihai Podgoreanu^2^, Quintin Quinones^2^

#### ^1^University College Dublin Medical School, Dublin, Ireland; ^2^Systems Modeling of Perioperative Cardiovascular Injury Laboratory, Department of Anesthesiology, Duke University, Durham, NC, USA; ^3^Division of Surgical Sciences and CFAR, Department of Surgery, Duke University, Durham, NC, USA; ^4^Institute of Arctic Biology, University of Alaska at Fairbanks, Fairbanks, AK, USA

##### **Correspondence:** James Griffin

**Introduction:** As part of a greater project investigating the natural immunomodulation witnessed in hibernating animals we needed to establish a comparative in non-hibernating animals. This is the first step in a group of experiments to allow insight into the differences in the action of the immune systems of hibernators vs non-hibernators.

**Methods:** Using a lentiviral vector, Rat Macrophage CLR-2192 cells were produced expressing GFP and luciferase. Differing concentrations of Polybrene, DEAE Dextran and Cyclosporine A were utilized to attempt to aid the transduction of the virus at 50, 40 and 25 MOIs. GFP expression was monitored daily using florescent microscopy. Supernatants were taken on varying time-courses to test for secreted luciferase; cells were also lysed using a lysis buffer (Luc-lysis Buffer, GeneCopoeia); luciferase assay was then performed on these samples, using a luminator, and the results assessed.

**Results:** Our results clearly demonstrated a greater transduction with the DEAE Dextran than with the virus alone or either of the other transduction aids. The most effective DEAE Dextran concentration appears to be 6ug/mL with an RLuc of 50 MOI producing a max Relative Light Unit (RLU) reading of 88,575 at 144h. Compare this to the max RLU reading for CSA at 8,634 at 144h at 50 MOI and max reading for purely virus 29,443 at 40 MOI 120h.

**Discussion:** We have produced an effective transduction protocol for these rat macrophage cells. We shall use this to further investigate the immunoregulation of hibernating animals and how to make a difference in the clinical setting in the future.

## P7 The incidence of cancer in Bosnia and Herzegovina and Croatia - comparison with other European countries

### Marin Lakic^1^, Kristian Karlovic^1^, Ante Rotim^2^, Jerko Vicic^1^, Jelena Ravlija^3^, Krunoslav Capak^4^

#### ^1^Faculty of Medicine, University of Mostar, Mostar, Bosnia and Herzegovina; ^2^Faculty of Medicine, University of Rijeka, Rijeka, Croatia; ^3^Epidemiology, Institute for Public Health FBiH, Mostar, Bosnia and Herzegovina; ^4^Epidemiology, Croatian Institute of Public Health, Zagreb, Croatia

##### **Correspondence:** Kristian Karlovic

**Introduction:** It’s known that the worldwide incidence of colorectal cancer and prostate cancer in man and breast cancer in women are on the first place. In the countries of Eastern Europe it’s a different situation. The aim of this study was to point out big public health problem, the incidence of lung cancer in Eastern Europe.

**Methods:** This is a retrospective study where we collected the data in period from 2008. to 2012. We’ve analyzed the incidence of lung cancer, colorectal cancer, prostate and breast cancer in Croatia and Federation of Bosnia and Herzegovina, and compared with Western European countries.

**Results:** From 26552 analyzed cases of cancer in the Federation of Bosnia and Herzegovina: 4751 were lung cancer, 2770 colorectal cancer, 2238 breast cancer, 1553 prostate cancer. In Croatia we found 103617 cases of cancer: 14489 colorectal cancer, 13787 lung cancer, 11815 breast cancer, 8645 prostate cancer. Considering the most risky factor (smoking) this research makes sense (WHO: 40,7% people in Bosnia and Herzegovina are smokers).

**Discussion:** In other European countries prostate cancer takes the first place in men, in Federation of Bosnia and Herzegovina third. First place is taken by lung cancer and second by colorectal cancer, in Croatia the first place in men is taken by lung cancer, second by prostate cancer and third by colorectal cancer. In both countries, the most common cancer in women is breast cancer, second placed is colorectal and third placed is lung cancer.

## P8 Burnout, physical activity and extracurricular activity in medical students

### Philip Macilwraith^1^, Deirdre Bennett^2^

#### ^1^School of Medicine, University College Cork, Cork, Ireland; ^2^Medical Education Unit, University College Cork, Cork, Ireland

##### **Correspondence:** Philip Macilwraith

**Introduction:** Burnout is common in medical professionals and can impact on patient care and career fulfillment. In medical students, burnout is associated with increased levels of stress and lower productivity; however, it’s unclear what factors contribute to its development. This study sets out to investigate whether physical activity levels predict burnout in medical students.

**Methods:** Medical students (n = 1004) at University College Cork completed either an online or a written questionnaire assessing: emotional exhaustion (EE), depersonalization (DP), personal accomplishment (PA), physical activity levels and extracurricular activity engagement.

**Results:** Approximately 45% (44.8%) of students reported high levels of EE, 26% (25.6%) reported high levels of CY, and 51% (51.2%) reported low levels of AE. 53.2% of respondents were found to be HEFA active (Health-enhancing physical activity). Analysis of variance revealed significant effects of gender, year group, nationality and levels of physical activity and the three components of burnout (EE, CY and AE). Using the bivariate Pearson correlation, it was found that there was a correlation between AE and physical activity levels (0.134, p < .005 for a two-tailed test). The most commonly engaged in extracurricular activities were going to the gym (30.2%) and athletics (19.9%).

**Discussion:** Burnout is present in medical students in University College Cork, and levels of physical activity predict certain components of burnout. Gender, year group and nationality group also appear to influence the prevalence of burnout and physical activity levels. Encouraging medical students to engage in health-enhancing physical activity early in their medical training may reduce the likelihood of developing burnout.

## P9 Incidence of postoperative nausea and vomiting in caesarean section patients and the clinical utility of antiemetics in this cohort

### Celine Meschino^1^, Fergus Walsh^2^

#### ^1^Graduate Entry Medicine, University College Cork, Cork, Ireland; ^2^Anaesthesia, Cork University Hospital, Cork, Ireland

##### **Correspondence:** Celine Meschino

**Introduction:** The reported incidence of post-operative nausea and vomiting (PONV) following caesarean sections (C-sections) varies from 15-80%. The incidence PONV in this cohort is unknown at Cork University Maternity Hospital (CUMH). An audit of PONV after C-sections was completed at CUMH to determine the incidence of PONV and the factors associated with it.

**Methods:** The incidence of PONV was recorded and clinically significant PONV evaluated using the validated Postoperative Nausea and Vomiting Intensity Scale, in 55 post C-section patients at CUMH at time intervals of 0-6 and 6-24 hours postoperatively. Patient characteristics and intraoperative pharmacological interventions were collected from patient medical records. Pearson’s Chi-Squared Independence Tests were used to determine the association between PONV and patient characteristics and intraoperative pharmacological interventions. Independent T tests and a Mann-Whitney U test were used to determine the association between PONV and patient age, intraoperative phenylephrine dose and BMI respectively.

**Results:** Between 0-6 hours postoperatively 38.2% of participants experienced PONV and16.4% experienced clinically significant PONV. From 6-24 hours 30.9% of participants experience PONV and 9.1% experienced clinically significant PONV. Intraoperative antiemetic administration was significantly associated with no PONV 0-6 hours post C-section (p = 0.020). Furthermore, significantly higher phenylephrine doses were administered to patients that experienced clinically significant PONV between 0-6 hours (p = 0.003).

**Discussion:** The incidence of PONV after C-section at CUMH is greater than 30% at 0-6 and 6-24 hours postoperatively. This is of particular importance in this study group due to the added responsibilities of motherhood during the postoperative period in addition to recovery from major surgery.

## P10 A study of Placental Growth Factor levels in twin pregnancy and their use as a prognostic tool for adverse outcomes

### Caoimhe Normile^1^, Deirdre Hayes-Ryan^2^, Eve O’Mahony^1^, Louise Kenny^3^

#### ^1^School of Medicine, University College Cork, Cork, Ireland; ^2^INFANT Research Centre, University College Cork, Ireland; ^3^Department of Obstetrics and Gynaecology, Cork University Maternity Hospital, Cork, Ireland

##### **Correspondence:** Caoimhe Normile

**Introduction:** Placental growth factor (PlGF) is a biomarker which has been shown to be much reduced in pre-eclampsia, and has demonstrated sensitivity and specificity in its diagnosis. Pre-eclampsia occurs in 2-8% of pregnancies and is 2-3 times more likely in twin pregnancy. Pre-eclampsia increases maternal morbidity and mortality, and causes 15% of preterm births. Use of singleton pregnancy reference ranges for PlGF is inappropriate due to their smaller placental mass. Primary aim of this study is to develop a twin pregnancy specific reference range for PlGF. Secondary aim is to assess whether there is a correlation between low PlGF values and hypertensive disorders of pregnancy (HDP).

**Methods:** This is an on-going prospective cross sectional study of consenting women between 12 and 36 + 6 weeks gestation with a twin pregnancy. Basic demographic details are recorded at recruitment and a blood sample is taken, centrifuged and plasma stored at -8°C for later plasma quantification of PlGF by immunoassay. Postnatal pregnancy outcome data is obtained by chart review.

**Results:** 163 women have been recruited to date, with PlGF analyzed in 24. PlGF levels in twin pregnancy peak at 32 weeks with a subsequent decline until term. The median PlGF in those with HDP was 82.2pg/ml compared to 252pg/ml in those without, however this difference was not statistically significant.

**Discussion:** PlGF values in twin pregnancies follow a similar pattern as described previously for singletons and tracks placental development. Analysis of PlGF in remaining samples is needed to assess for correlation between low PlGF values and HDP.

## P11 An analysis of paediatric RAST tests at a district general hospital

### Aishan Patil^1^, Anish Patil^2^, Reza Saeidi^3^, Nash Patil^3^

#### ^1^Medicine, University of Glasgow, Glasgow, UK; ^2^School of Medicine, Royal College of Surgeons in Ireland, Dublin, Ireland; ^3^ENT, Sligo University Hospital, Sligo, Ireland

##### **Correspondence:** Aishan Patil

**Introduction:** A study was conducted at Sligo University Hospital by extracting data on RAST tests undertaken on a paediatric patient population who attended the ENT and paediatrics units with symptoms suggestive of allergic rhinitis.

**Methods:** A total cohort of 98 children who were tested in the first half of 2014 was included in the database. Their demographics were computed, including average age at presentation, and gender ratio. RAST tests at the hospital looked at the most common allergens such as house dust mites, timothy grass, wheat, egg white and milk. However, a number of less common allergens were included such as soya, tomato and apple. The study looks at the outcomes of RAST testing in this patient population and attempts to correlate these findings with environmental allergens found in the north-west of Ireland.

**Results:** House dust mite was the most common allergen in the cohort, with 57 (58% of the cohort) patients showing some immunological response, followed by milk with 55 (56%) patients. 31 children were allergic to cat dander. Some uncommon allergens such as orange (2 patients), penicillin G (2 patients) and pea (1 patients) were also noted.

**Discussion:** Our study, it is hoped, will allow an assessment of the specificity and sensitivity of the test as it relates to this part of the world. Sligo University Hospital has commenced a skin allergy testing service in the last year or so, and it is hoped that future data will be able to give us a better idea of the best way forward for investigating this common condition in the paediatric age group.

## P12 Sensitivity And Specificity Of MRI Scanning In Epilepsy - A Retrospective Review

### Aishan Patil^1^, Mosab Mahadi^2^, Kevin Murphy^3^

#### ^1^Medicine, University of Glasgow, Glasgow, UK; ^2^ENT, Sligo General Hospital, Sligo, Ireland; ^3^Neurology, Sligo General Hospital, Sligo, Ireland

##### **Correspondence:** Aishan Patil

**Introduction:** At present, epilepsy affects over 40,000 in Scotland (and up to 65 million worldwide), making it the 5th most common neurological condition in the UK.

Diagnostic imaging techniques include MRI and PET.

Some of the findings on neuroimaging in those with medically uncontrollable epilepsy include epileptogenic lesions, subcortical white matter hyper-intensities, cavernous malformations, and occasionally brain tumours.

**Methods:** As outlined in the abstract, amongst factors influencing the need for surgical management of epileptic patients is accurate localization of a lesion using diagnostic imaging. Conventional imaging techniques for identifying such lesions in current practice are MRI and PET, and therefore our aims were to document a presence of any MRI findings in epileptic patients.

This submission analyses the demographics of sixty patients who underwent an MRI brain (epilepsy protocol) at a district general hospital. Their findings are assessed, and the sensitivity of the test is analyzed. Whether such imaging plays a role in determining treatment or management plans is debated.

We chose to carry out a closed retrospective study using patient notes of sixty diagnosed epileptics over a period of five years) in a single hospital, documenting our findings as listed above. Other factors we documented and took into consideration was the type of epilepsy, if diagnosed, any EEG findings if recorded, known other medical conditions or medications, lifestyle aspects as relevant, the presence of a family history and the history of their diagnosis.

**Results:** Findings are presented in a tabulated fashion. Overall the test does not seem to have a high degree of sensitivity.

**Discussion:** MRI brain scanning in this cohort of patients needs to be a part of a test battery in order to give effective clinical information. In of itself, it does not appear to have a significant value as a stand-alone investigation.

## P13 Correlation between morphological structure and efficiency of restoration of immune function of splenic implant in patients after splenectomy

### Olga Smorodska, Volodymyr V. Shevchenko, Volodymyr P. Shevchenko

#### Department of General Surgery, Medical Institute of Sumy State University, Sumy, Ukraine

##### **Correspondence:** Olga Smorodska

**Introduction:** The most common method of surgical treatment of severe spleen injuries is splenectomy (SE). SE causes serious immune disorders to correct which helps saving of functioning splenic tissue by its autotransplantation. For the immune function(IF) corresponds white pulp (WP), which distribution is uneven in the spleen. The aim is to study the dependence of efficiency of restoring of IF of splenic implant on its morphological structure.

**Methods:** Histological investigations of different parts of splenic tissue for determination of the largest concentration of WP elements (concentration of Malpighian cells and clusters of lymphoid tissue) were made. Coloring of micropreparations by Romanovskyy -Himza was used. Investigations were conducted on cadaveric spleen after its laundering with hypertonic salt solution for complete removing of red blood cells. Immune status of patients evaluated by CD3, CD4, CD8, Ig A, M, G, NK, which were determined on 7,30 day and 3 months after SE. Average indicators of healthy individuals (donors) were used as a control.

**Results:** It was found that the largest concentration of WP was in areas which are located in 6-8 mm from the capsule. Considering obtained results for autotransplantation were used tissues from subcapsular area. First 7 days after SE wes recorded a significant reduction in all investigated indicators, which began to rise up after 30 days and after 3 months did not differ from relevant indicators of donors (P >0.05).

**Discussion:** To improve the efficiency of autotransplantation it is advisable to use splenic tissue that containing the largest concentration of WP.

## P14 Likelihood of woman with a history of caesarean section to give a safe vaginal birth

### Kristians Suspanovs, Margarita Pukite

#### Faculty of Medicine, University of Latvia, Riga, Latvia

##### **Correspondence:** Kristians Suspanovs

**Introduction:** The number of ceasarean section (CS) performed in a group of women with CS history is increasing, while the number of vaginal birth (VB) in this group decreases. This study aims at examining the most common risk factors, concluding which of them affect VB after CS history, finding out the most common acute CS indication and investigating the probability of safe VB after CS.

**Methods:** Data included in outpatient cards were analysed in a descriptive study. The study took place in Jelgava regional hospital and the data were collected during the period 01/2013-12/2015. The study analysed data of 74 women who tried to give birth vaginally after CS history. The obtained data were analysed with MS Excel and IBM SPSS.

**Results:** Out of 74 VB after CS history, 67 of which ended in VB and 7 in acute CS. Uterine dysfunction -- most frequent acute CS indication (n = 3; 43%). Most respondents had one CS (n = 70; 94.59%) and two CS (n = 4; 5.41%). Average age: 30.13 +/- 4.8 years. Average BMI = 24.05 +/- 4.35 (kg/m2). Average newborn birth weight = 3479.05 +/- 360.84 (g). Average thickness of the uterine scar = 3.93 (mm). The most used induction method was with oxytocin (n = 32/55; 43.24%).

**Discussion:** Women with CS history are safe to have VB 90.55%. Acute CS indication in 43% case - uterine disfunction. The most common risk factor was not found, but it is known that certain risk factors have risks associated with them and may adversely affect VB outcome.

## P15 Efficacy of ombitasvir/paritaprevir/ritonavir & dasabuvir with/without ribavirine in the treatment of Hepatitis C (1b) infected cirrhotic patients

### Joseph Abuazza^1^, Abir Abuazza^2^, Mohamed Abuazza^2^, Irina Girleanu^3^

#### ^1^Gastroenterology & Hepatology, Grigore T. Popa University of Medicine and Pharmacy, Iasi, Romania; ^2^Grigore T. Popa University of Medicine and Pharmacy, Iasi, Romania; ^3^Gastroenterology & Hepatology, Grigore T. Popa University of Medicine and Pharmacy, St Spirdon Hospital, Iasi, Romania

##### **Correspondence:** Joseph Abuazza

**Introduction:** Chronic hepatitis C virus Infection and cirrhosis have a higher risk for liver-related complications. This study assessed the efficacy and safety of ombitasvir/paritaprevir/ritonavir and dasabuvir, without ribavirin, for 12weeks in HCV genotype 1b infection and compensated cirrhosis patients.

**Methods:** Peginterferon/ribavirin-treated patients prospectively received 12weeks of ombitasvir/paritaprevir/ritonavir and dasabuvir. Treatment Criteria: Stage 4 fibrosis evaluated by Fibromax, compensated cirrhosis (maximum Child A6), with or without previous decompensating, no evidence of hepatocellular carcinoma, sober >6 months, with no age limit.Efficacy was assessed by the percentage of patients achieving Sustained Virological Response (HCV RNA undetectable) 12weeks post-treatment.

**Results:** Between December 2015-February 2016, 196 patients were analyzed. 126 (64.3)% female, 54.05% treatment-experienced, Child-Pugh A5 class (76.8%), 24.3% with esophageal varices, and 5.3% with albumin <3.5g/dl. The overall rates EOT were 96.4% for ITT. ITT analysis showed negative PCR at the SVR12 in 95.9% of cases and 99.4% per protocol.

Treatment discontinuation rate was 3.58% (7 patients)-1 depression, 2 cardiac failure, 1 cardiac arrhythmia, 1 acute kidney failure, 1 hepatic encephalopathy, 1 acute liver failure.

Adverse events were: anemia (33.8%), asthenia (25.2%), insomnia (15.6%), and pruritus (13.9%). Two patients died from cardiovascular disease and acute liver failure. During the follow-up period, three patients developed variceal bleeding.

**Discussion:** The HCV regimen of ombitasvir/paritaprevir/ritonavir and dasabuvir with or without ribavirin for 12 weeks had a high efficacy and was well tolerated in HCV genotype 1b-infected patients with cirrhosis.

## P16 STAT3 siRNA loaded Aptamer-PCB co-modified liposomes for lung cancer targeted delivery

### Fatema Ahmed Ali^1^, Jing-Hao Cui^2^

#### ^1^College of Medicine, RCSI-MUB, Muharraq, Kingdom of Bahrain; ^2^Collage of Pharmaceutical Sciences, Soochow University, Suzhou, China

##### **Correspondence:** Fatema Ahmed Ali

**Introduction:** Aptamers are single stranded DNA or RNA that can bind to pre selected targets. AS1411 aptamer is an antinucleoline aptamer, targeting the protein nucleolin, which is over expressed on the surface of certain cancer cells. STAT3 is a signal transducer and a transcription factor, involved in cell cycle regulation. Its over activation result in oncogenesis, uncontrolled proliferation and resistance to anti-cancer drugs. This project aims at achieving targeted therapy to lung cancer cells by co-modifying PCB liposomes via AS1411 aptamers and using inhalation as a route of delivery.

**Methods:** PCB liposomes were formed that and conjugated with NH2-Apt. They were then loaded with STAT3 siRNA and introduced to A549, NSCLC cells. After testing for successful genetic silencing, the effect of genetic silencing on apoptosis and sensitivity to DDP were tested. The effect of STAT3 gene silencing was tested in vivo in tumor bearing nude mice by evaluating its effect on tumor size.

**Results:** It was found that treatment with inhaled STAT3 Apt-CELs and IV DDP resulted in a significantly smaller tumour size over the course of treatment although, cellular apoptosis was not very well achieved.

**Discussion:** Apt-CELs were successfully prepared, with high cellular uptake and in vivo distribution. In addition, they were effectively delivered to the lungs and STAT3 gene silencing was achieved in tumour bearing nude mice. However, its effects on cell apoptosis after cellular uptake of STAT3-siRNA were not as efficient compared with liposomes that are commercially available (Lipofectamine 2000). The study results provide evidence that STAT3 enhances cellular sensitivity to DDP and that using AS1411 Apt-CELs via inhalation increased the concentration of delivered modified liposomes to the lungs.

## P17 Predisposing factors, symptoms and after-effects related with nausea and vomiting in gastric cancer patients

### Daniel Andrysiak^1^, Iwona Filipczak-Bryniarska^2^, Jakub Kucharz^3^

#### ^1^Clinic of Pain Research and Management, Jagiellonian University Medical College, Cracow, Poland; ^2^Clinic of Pain Research and Management, The University Hospital in Cracow, Cracow, Poland; ^3^Department of Experimental and Clinical Surgery, Institute of Physiotherapy, Faculty of Health Sciences, Jagiellonian University Medical College, Cracow, Poland

##### **Correspondence:** Daniel Andrysiak

**Introduction:** Nausea and vomiting are significant symptoms in cancer patients, especially cancer of the digestive system. Goal of our research was to define the predisposing factors, symptoms and after-effects of nausea and vomiting at admission.

**Methods:** A retrospective analysis was performed on 110 gastric cancer patients admitted to the Palliative Care Unit. We analyzed data of the physical examination, medical history, along with the lab results (blood cell count, albumin, sodium, potassium, calcium, LDH, hemoglobin). Univariate and multivariate analysis were used to define the predisposing factors, symptoms and after-effects of nausea and vomiting at admission.

**Results:** 50% of patients had nausea and vomiting at admission. These patients were more likely to bone metastases (CI95%:0.011-0.7753, OR = 0.0946,p = 0.02), constipation at admission (CI95%:1.5177-7.2441, OR = 3.3157,p = 0.0026), constipation during hospitalization (CI95%:1.2181-5.7048, OR = 2.6361,p = 0.0138), hypokalemia (CI95%:0.2345-0.7185, OR = 0.4105,p = 0.0018), decreased hemoglobin (CI95%:1.053-1.6494, OR = 1.3179,p = 0.015), and used GKS (CI95%:1.0498-5.8141, OR = 2.4705,p = 0.0383) (univariate logistic regression analysis). Multivariate logistic regression analysis shows that the factors related to nausea and vomiting are constipation at admission (CI95%:1.6626-15.8167, OR = 5.128,p = 0.004), bone metastases (CI95%: 0.006-0.9436, OR = 0.0756,p = 0.045), anxiety at discharge from hospital (CI95%: 0.0483-0.9311, OR = 0.2121,p = 0.04) and a shorter hospitalization period (CI95%: 0.8845-0.9869, OR = 0.9343,p = 0.015).

**Discussion:** Nausea and vomiting at admission are connected with the following factors: bone metastases, constipation, hypokalemia, lower hemoglobin concentration, the use of corticosteroids, a shorter period of hospitalization and anxiety. Nausea and vomiting are serious problem in patients suffer from gastric cancer significantly decreasing the quality of life, so it is very important to protect from these symptoms in cancer patients.

## P18 Suicide attempts among opiates addicts

### Jasna Bojovic, Aleksandra Dickov

#### Department of Psychiatry, University of Novi Sad, Novi Sad, Serbia

##### **Correspondence:** Jasna Bojovic

**Introduction:** Suicide is series of events that result in taking one persons own life, driven by the need to end his own life, in which case he is aware of his actions. Suicide is closely linked to the substances use.Opiates divided in four categories: morphine, codeine, heroin and dilaludin. Opiods (methadone, buprinorphine) they are working through opioids receptors and they are used for treatment of opiates addicts.

**Methods:** The subjects have been divided into two groups: a control group consisting 30 healthy volunteers, and an experimental group of 30 opiates addicts, clients of Methadone clinic center in Novi Sad. Columbia suicide severity rating scale- screening version (C-SSRS) was used to determine suicide rate.

Goal: 1.To determine the existence of a difference between aquired with the suicide assessment scale (C-SSRS) and socio-demographic questionnaire on opiates addicts 2. To determine the existence of a difference between aquired with the suicide assessment scale (C-SSRS) and socio-demographic questionnaire on healthy control group 3. To determine the existence of a difference comparing scores between aquired with the suicide assessment scale (C-SSRS) and socio-demographic questionnaire between the groups of opiates addicts and healthy control group.

**Results:** Between opiates addicts and healthy volunteers is noticed statistically significant difference in results in suicide attempts(x(1) = 4.812, p = 0.028), social financially depended (x(1) = 4.593, p = 0.032), employment status (x(1) = 6.944, p = 0.008).

**Discussion:** Suicide attempts are more distinctive in opiates addicts group than in group of healthy volunteers, social and financially dependent subjects are more distinctive in opiates group than in group of healthy volunteers, as same as unemployment.

## P19 Factors associated with dyspnea in patients with stomach cancer

### Pawel Bryniarski^1^, Iwona Filipczak-Bryniarska^2^, Jakub Kucharz^3^

#### ^1^Departement of Pain Treatment and Palliative Care Students Scientific Group, Jagiellonian University Medical College, Krakow, Poland; ^2^Department of Palliative Care, Jagiellonian University Medical College, Krakow, Poland; ^3^Department of Experimental and Clinical Surgery, Institute of Physiotherapy, Faculty of Health Sciences, Jagiellonian University Medical College, Krakow, Poland

##### **Correspondence:** Pawel Bryniarski

**Introduction:** Dyspnea is a common and serious problem in patients with terminal stomach cancer. The aim of our study was to determine the predictors, symptoms and consequences of dyspnea.

**Methods:** 110 terminal stomach cancer patients admitted to Palliative Care Unit were retrospectively analyzed. Detailed physical examination, medical history including history taken from family and care givers was taken upon admission. Laboratory parameters including morphology, sodium, potassium, total and ionized calcium, LDH were taken on admission. We used univariate and multivariate logistic regression analysis to determine possible predictors, symptoms and consequences of dyspnea.

**Results:** On admission 26,36% of patients had dyspnea. They had more often bone metastases (OR = Odds Ratio = 5,022, CI95% = Confidence Interval 95% = 1,304-19;336 p = probability value = 0,019), had more often weightloss > 30% (cachexia) (OR = 6,594, CI95%:1,846-23,561; p =0,0037), had higher levels of potassium (OR = 1,744, CI95%:1,027-2,963; p = 0,0397) and more often oxygen therapy (OR = 5,008, CI95%:1,652-15,179; p = 0,0044). Multivariate logistic regression analysis after adjustment for possible confounders reviled that weightloss > 30% (OR:11,304, CI95%:2,112-60,497; p = 0.005), bone metastases (OR:40,416, CI95%:4,753-343,67; p = 0,002), shorter duration of treatment (OR:0,923, CI95%:0,865-0,985; p = 0,015, because of the higher risk of death) and lower use of opioids (OR:0,162, CI95%:0,037-0,703; p = 0,015) remained independently associated with dyspnea.

**Discussion:** Cachexia, bone metastases, shorter duration of treatment and lower use of opioids are risk factors of dyspnea. The serious condition of stomach cancer patients are connected with bad prognosis of their survival. Results of our study determine that dyspnea is a serious clinical symptom at the last stage of stomach cancer worsens the survival of patients with cachexia.

## P20 Prognostic factors, symptoms and consequences of dyselectrolytemia in terminal cancer patients

### Pawel Bryniarski^1^, Iwona Filipczak-Bryniarska^2^, Jakub Kucharz^3^

#### ^1^Departement of Pain Treatment and Palliative Care Students Scientific Group, Jagiellonian University Medical College, Krakow, Poland; ^2^Department of Palliative Care, Jagiellonian University Medical College, Krakow, Poland; ^3^Department of Experimental and Clinical Surgery, Institute of Physiotherapy, Faculty of Health Sciences, Jagiellonian University Medical College, Krakow, Poland

##### **Correspondence:** Pawel Bryniarski

**Introduction:** Dyselectrolytemia is a common problem in patients with terminal cancer. It worsens the quality of life and increase the amount of complications. The aim of our study was to determine prognostic factors, symptoms and consequences of dyselectrolytemia.

**Methods:** 242 terminal cancer patients admitted to Palliative Care Unit were retrospectively analyzed. Detailed physical examination, medical history including those delivered by family and care givers was taken upon admission. Laboratory parameters including morphology, sodium, potassium, total and ionized calcium blood concentration, LDH were taken on admission. We used univariate and multivariate logistic regression analysis to determine possible factors associated with dyselectrolytemia.

**Results:** On admission 61,98% of patients were diagnosed with dyselectrolytemia. They were 2,7x (2,7 times) more often admitted to the Palliative Care Department from Emergency Department, they had 1,6x higher score in PS scale note, they were 1,9 times more often cachectic. They died 2,7x more often than patients without dyselectrolytemia. Medical doctors gave them 5,94x more often intravenous fluids than for patients without this problem. They had 1,12x lower levels of sodium (OR = 0,892) and potassium (OR = 0,887). Patients with dyselectrolytemia had 7,5x more often metastases to the central nervous system. They were treated 2,14x more often with opioids.

**Discussion:** Admission from the Emergency Department to Palliative Care Unit, CNS metastases, prior opioid use administration are independently associated risk factors of dyselectrolytemia. Intravenous fluids supply, lower level of sodium and potassium are independently associated symptoms of dyselectrolytemia. Dehydration and death are independently associated consequences of dyselectrolytemia.

## P21 The influence of postoperative biliary occlusion on patients’ mean survival time after implantation of endoscopic stent

### Dorota Dlugosz, Monika Orlowska

#### Medicine, Jagiellonian University Medical College, Cracow, Poland

##### **Correspondence:** Dorota Dlugosz

**Introduction:** Endoscopic stents are commonly referred to as self-expandable metallic stents (SEMS). They play a significant role in the management of malignant obstructing lesions in the gastrointestinal tract. A frequent complication of this procedure is occlusion of the bile duct. Our objective was to determine what is the postoperative life expectancy of patients and what impact do complications have on the patients’ survival time.

**Methods:** Descriptive and retrospective analysis of 165 patiens who underwent an implantation of endoscopic stent due to biliary occlusion because of pancreatic or biliary malignancy. 94 of them were women. Patients were followed up in terms of stent occlusion in the postoperative course. Overall survival time was also assesed.

**Results:** Mean survival of the patients was 163 days. 21 patients had stent occlusion in the postoperative course. There were no statistical differences between mean survival time in the stent occlusion group and no-complication group, however it was higher in no-complication group (107 days vs 170 days, p = 0,08). There were no statistical differences between men and women regarding overall survival (p = 0,047).

**Discussion:** Self-expandable stents are efficient and effective way of paliative treatment of biliary occlusion due to malignancy. Stent occlusion in the postoperative course does not affect overall survival time.

## P22 Simultaneous inhibition of FLIP and MEK1/2 triggers apoptosis in V600E BRAF-mutant colorectal cancer models

### Emer Gates, Sandra Van Schaeybroeck, Daniel Longley

#### Centre for Cancer Research and Cell Biology, Queen’s University Belfast, Belfast, Northern Ireland

##### **Correspondence:** Emer Gates

**Introduction:** The BRAFV600E oncogenic mutation (BRAFMT) occurs in 8-15% of colorectal cancers (CRC) and confers a poor prognosis. Treatment with MEK1/2 inhibition in BRAFMT CRC cells activates the c-MET-STAT3 pathway. This promotes expression of FLIP, which inhibits caspase-8-mediated apoptosis. FLIP therefore promotes resistance to MEK1/2 inhibition in BRAFMT CRC cells.

Hypothesis: Treatment with inhibitors of FLIP will sensitise BRAFMT CRC cells to the MEK1/2 inhibitor AZD6244, resulting in increased apoptosis.

**Methods:** Cell lines: BRAFV600E LIM2405 and HT-29; isogenic BRAFV600E VACO432 and wild-type VT1. Experiments: MTT assays, Western blotting, flow cytometry, caspase activity assays, combination index (CI) assays (Calcusyn software), siRNA reverse transfection.

**Results:** BRAF wild-type (BRAFWT) cells have higher sensitivity to inhibitors of FLIP compared to BRAFMT cells, and this is due to induction of apoptosis as shown by PARP and caspase-3/7 cleavage. However, in BRAFMT cells, inhibition of FLIP results in hyper-sensitivity to MEK1/2 inhibition, with synergy observed in cell viability assays and supra-additive effects on apoptosis. We have shown that this synergy is dependent on caspase-8, consistent with FLIP inhibition being responsible for the observed effects. Despite high sensitivity to FLIP inhibition in BRAFWT cells, they are resistant to MEK1/2 inhibition, so the combination of FLIP and MEK1/2 inhibition is ineffective in these cells.

**Discussion:** This study confirms our hypothesis: inhibition of FLIP sensitises BRAFMT CRC cells to MEK1/2 inhibition. Combined inhibition of FLIP and MEK1/2 is therefore a potential treatment strategy for BRAFMT CRC, with the potential to enhance the poor survival rates in this disease subgroup.

## P23 Bioluminescent Monitoring of Rat Cardiac Stromal c-Kit(+) Cell in Platelet Gel Engraftment in Ischemic Heart

### Elizaveta Milevskaia^1^, Sophia Pavlova^2^

#### ^1^Department of Medicine, Novosibirsk State University, Novosibirsk, Russia; ^2^Department of Developmental Epigenetics, Institute of Cytology and Genetics, Novosibirsk, Russia

##### **Correspondence:** Elizaveta Milevskaia

**Introduction:** Cell technology is one of the promising strategies for ischemic heart injury treatment. The major point of success is high efficiency of cell delivery and homing. We have suggested that the use of platelet gel contributes to better engraftment and survival of transplanted cells.

**Methods:** A cardiac stromal c-Kit-positive (CSC) cell culture, which has features of mesenchymal stromal, endothelial cells and an angiogenic potency, was derived by cardiosphere method. CSC’s were transplanted intramyocardially to peri-infarct zone after occlusion of the LAD artery. Cells were suspended in culture medium (group I) and in platelet plasma (group II) to improve attachment. Cells were modified by transduction of a lentiviral vector pLentiPGK V5-LUC Neo carrying luciferase and geneticin resistance genes. Luciferase activity in cell extracts of myocardium was assessed on the Wallac 1420 multilabel counter.

**Results:** A direct correlation between CSC-Luc number and luciferase activity allowed us to assess CSC-Luc engraftment quantitatively. Right after the transplantation luminescence of tissue extracts corresponds 42,9 ± 4,63% in group I and 38,31 ± 4,16% in group II. In 48 hours the number of cells increased in both groups. CSC-Luc’s were eliminated from myocardium of group I animals in 5 days, group II - in 10 days after the transplantation.

**Discussion:** Platelet gel has a positive influence on CSC-Luc engraftment. CSC-Luc’s proliferate during 48 hours after the transplantation. Period of cell survival corresponds to the acute stage of myocardial infarction when any impact on cardiomyocyte survival has a direct relevance to patient’s outcome.

## P24 The influence of ratio PLT/WBC on the complications and recurrence rate in bleeding from the upper and lower Gastrointestinal Tract (GI)

### Monika Orlowska, Marcin Bylina, Klaudiusz Bolt

#### Students’ Scientific Group at 2nd Department of Surgery, Krakow, Poland

##### **Correspondence:** Monika Orlowska

**Introduction:** Bleeding from the gastrointestinal tract represents a high percentage of diseases on the surgical wards. Consequently, methods of rapid and effective analysis are sought, which would allow for early prevention and to minimize the percentage of complications and relapses.

**Methods:** 223 patients were enrolled to the study (mean age 67), 137 showed bleeding in the upper GI tract and 86 in lower GI tract. We compared the results of blood count before and during hospitalization among patients of the 2nd Department of General Surgery JU MC in Krakow, who showed bleeding from the gastrointestinal tract. We took into consideration the ratio of PLT/WBC, recurrences of bleeding, complications and mortality.

**Results:** 16 (N = 7,17%) complications, 16(N = 7,17%) recurrences and 1 death occurred. The ratio of PLT/WBC in patients with relapse or complications is similar to the ratio of PLT/WBC in patients who showed no such complications (p = 0,64). Patients with upper GI bleeding have a lower ratio of PLT/WBC at the time of admission than patients with bleeding lower GI bleeding (p = 0.00). The PLT/WBC ration was similar on the day of patient’s discharge from the hospital (p = 0,36).

**Discussion:** The ratio of PLT/WBC is lower in the case of bleeding from the upper GI tract and may be useful in the diagnosis of bleeding site. However, the ratio occurred to be insignificant in terms of the incidence of recurrences, complications and mortality.

## P25 Anxiety and depression in cervical dystonia patients

### Denis Pap, Aleksandar Jesic

#### Department of Neurology, Clinical Center of Vojvodina in Novi Sad, University of Novi Sad, Novi Sad, Serbia

##### **Correspondence:** Denis Pap

**Introduction:** Cervical dystonia is the most common form of focal dystonia. Beside pain, psychiatric disorders such as anxiety and depression may impact clinical course and quality of life.

**Methods:** We examined a group of 28 cervical dystonia patients, and a control group of 17 blepharospasm patients. Clinical evaluation comprised age, disease duration, botulinum toxin treatment duration and subjective assessment of therapeutic effect. Toronto Western Spasmodic Torticollis Rating Scale (TWSTRS) was used for measuring the severity of dystonia, and Beck’s Depression Inventory (BDI II) and Hospital Anxiety and Depression Scale (HADS) for anxiety and depression. Quality of life was determined by Craniocervical Dystonia Questionnaire (CDQ-24) in Serbian, which contains 5 subscales: Stigma, Emotional wellbeing, Pain, Activities of daily living, and Social/family life.

**Results:** Scores of anxiety measured with Hospital Anxiety and Depression Scale and depression with Beck’s Depression Inventory were significantly lower in the group of cervical dystonia. In the same group age conversely correlated with total Hospital Anxiety and Depression Scale. Stigma was worse in cervical dystonia, while Pain was not significantly different then in blepharospasm. Depression measured with Beck’s Depression Inventory correlated with all subscales of quality of life, while total Hospital Anxiety and Depression Scale score was the strongest predictor of quality of life.

**Discussion:** Cervical dystonia patients were significantly younger than blepharospasm patients, with lower scores of anxiety and depression. Stigma was worse in cervical dystonia. Depression correlates with all subscales of quality of life, and together with anxiety represents the strongest predictive factor of quality of life in cervical dystonia.

## P26 Polymorphism of ADRB2 gene is not associated with PTB: Analysis in Slovenian sample and Meta-analysis

### Ana Peterlin^1^, Ales Maver^1^, Ziga Jan^2^, Luca Lovrecic^2^, Natasa Tul^2^, Borut Peterlin^1^

#### ^1^Clinical Institute of Medical Genetics, University Medical Centre Ljubljana, Ljubljana, Slovenia; ^2^Department of Perinatology, University Clinical Centre Ljubljana, Ljubljana, Slovenia

##### **Correspondence:** Ana Peterlin

**Introduction:** The beta-2- adrenergic receptor (ADRB2) gene has an important impact on smooth muscle relaxation, including the smooth muscles of the uterus. The results of previously published studies of the association between ADRB2-rs1042713 polymorphism and spontaneous preterm birth (SPTB) were inconsistent.

**Methods:** We evaluated the association between ADRB2 and SPTB in a case-control association study of Slovenian sample and performed meta-analysis of previously published studies.

**Results:** No association was found between the variation of ADRB2 and SPTB in Slovenian sample of 112 SPTB patients and 143 controls under dominant (χ^2^ = 0.03, P = 0.86, OR = 1.05, 95% CI = 0.62-1.77), recessive (χ^2^ = 0.09, P = 0.77, OR = 0.91, 95% CI = 0.46-1.76) and co-dominant genetic models (χ^2^ = 0, P = 0.96, OR = 0.99, 95% CI = 0.6-1.62). Meta-analysis of a pooled sample of 428 SPTB patients and 886 controls suggested no association of *ADRB2* polymorphism and SPTB under dominant (OR = 0.84, 95% CI = 0.64-1.1) and recessive genetic models (OR = 0.65, 95% CI = 0.37-1.14).

**Discussion:** These findings suggest no firm association between polymorphism in ADRB2 gene and SPTB. Further association studies with large sample sizes are needed.

## P27 The effects of Aβ42 and α_1_-adrenergic receptor antagonists on α_1_-adrenergic receptor expression in cortical neurons

### Joanne Ridgley, David Whitfield, Jonathan Corcoran

#### Wolfson CARD, King’s College London, London, UK

##### **Correspondence:** Joanne Ridgley

**Introduction:** Currently, no licensed disease modifying therapies in Alzheimer’s Disease (AD) exist. Research continues to exploit different techniques to find a therapy, including analysis of genetic perturbations using the Connectivity Map Project. Analysis of this project suggests that α_1_-adrenergic receptor antagonists may yield therapeutic effects. This investigation aims to ascertain whether α_1_-adrenergic receptor antagonists can prevent Aβ42 mediated neuronal cell death.

**Methods:** Embryonic mouse cortical neurones were treated with doxazosin and HEAT hydrochloride (α_1_-adrenergic receptor antagonists) and Aβ42, individually and together along with a control. Immunohistochemistry and confocal microscopy was used to analyse cell death and α_1_-adrenergic receptor movement.

**Results:** Doxazosin was able to prevent Aβ42 mediated neuronal cell death. Doxazosin, HEAT hydrochloride and Aβ42 caused a reduction in the ratio of fluorescence of α_1_-adrenergic receptors in the neurite/cell body.

**Discussion:** Although doxazosin was able to prevent neuronal death in these in vitro assays, the assays lack many pathogenic features of AD. Thus, results may not translate into therapeutic action. Reduction in the ratio of receptors induced by doxazosin and HEAT hydrochloride may suggest that, in preventing neuronal death, they cause an internalisation of receptors, protecting them from extracellular Aβ42 interactions. Reduction in the ratio of fluorescence caused by Aβ42 alone is likely induced by a different mechanism, such as destruction of cell surface receptors, as Aβ42 is known to cause neuronal death. Cell surface biotinylation assays are needed to confirm these hypotheses. If results remain positive, these drugs could be used clinically to treat AD in future.

## P28 Violence among youth aged 15-18 on the territory of Autonomous Province of Vojvodina

### Mina Stanikic, Sanja Harhaji

#### Institute of Public Health of Vojvodina, Medical Faculty Novi Sad, University of Novi Sad, Novi Sad, Serbia

##### **Correspondence:** Mina Stanikic

**Introduction:** Since the first researches on violence among youth and violence in schools, studies on the topic are as important, and desirable, as they raise awareness among youth about the usage of violence. School violence is a regular phenomenon and is considered to be youth violence, while youth violence is not always school violence. Nonetheless, these two are tightly connected and permeate each other.

**Methods:** Database used for this research comes from the national study “Serbian population health research” conducted by the Ministry of Health in the last quartile of 2013. Specially designed questionnaire was applied as a research instrument. Used data is considering usage of and exposure to violence.

**Results:** This research included 164 examinees, aged 15, 16, 17 and 18 (high school age), of which 55,5% were women. All age groups were approximately equally present. Considering exposure to violence in schools in the last 12 months, 4,0% of examinees were exposed to physical violence, whereas 9,9% reported being exposed to psychological violence. There were no statistically significant differences between genders, except for the case of exposure to psychological violence in the street (male: 8,7%, female: 0%). Considering age groups, there was a significant difference in asking a teacher for help after being exposed to violence, where the 17-year-olds asked the most.

**Discussion:** The research has shown that psychological violence among older teenagers in Vojvodina is more present than physical, whereby the distribution of exposure to violence in school is about equal between genders and in all age groups.

## P29 Prevalence and risk factors of back pain among medical students in Bukovina Region of Ukraine

### Ivanna Yaremchuk, Olena Filipets, Oksana Yaremchuk

#### Department of Nervous Diseases, Psychiatry and Medical Psychology, Higher State Educational Institution, Bukovinian State Medical University, Chernivtsi, Ukraine

##### **Correspondence:** Ivanna Yaremchuk

**Introduction:** Back pain is among the most common conditions for which patients seek medical care. Identifying back pain risk factors is necessary for treatment and effective prevention. The aim of the present study was to research the prevalence and risk factors of back pain among Bucovina medical students.

**Methods:** A cross-sectional study was conducted in May 2016. 258 students of Bukovinian State Medical University aged 19 to 26 were interviewed by using specially designed questionnaires.

**Results:** Our study has found that back pain bothers 187 (72.5%) students. Among respondents periodic back pain (1-2 times a year) is observed in 64.7% (121 students). 45 (24.1%) students experience back pain 1-2 times a month, 11 (5.9%) students have chronic daily back pain that has bothered them more than one year and 10 (5.3%) students have acute back pain. Among girls the prevalence of back pain is higher than among men - 51.9% and 48.1% respectively. Among the students the most frequent pain (38.5%) is lower back pain, 30.5% of respondents have neck pain, 17.6% has middle back pain, combined pain is characteristic for 13.4%. According to the students’ responses the most prevalent causes of back pain are an excessive exercise: 27.8%, a prolonged stay in an uncomfortable position: 24.6%, trauma: 13.4% and the other causes: 34.2% of students.

**Discussion:** By means of the research we found high prevalence of back pain among Bukovinian medical students. This problem requires further study and active prevention of back pain to improve the quality of medical students’ life.

## P30 To examine the efficacy of insulin pump therapy in type 1 diabetes (T1DM) in a longitudinal retrospective study in Mater University Hospital Dublin

### Safari Aketch^1^, Alexa Resler^1^, Siobhan Bacon^2^, Maria Byrne^2^, Jochen Prehn^1^

#### ^1^Department of Physiology, Royal College of Surgeons in Ireland, Dublin, Ireland; ^2^Mater Misericordiae University Hospital, Dublin, Ireland

##### **Correspondence:** Safari Aketch

**Introduction:** Ireland uses insulin pump therapy sparingly despite the benefits that it can have for type 1 diabetes mellitus patients (T1DM).The aim in this study is to examine the clinical application and outcomes of pump therapy in T1DM patients.

**Methods:** A longitudinal retrospective chart review was used to evaluate patient outcome in T1DM that changed to pump therapy. Clinical data was obtained solely from Mater University Hospital (MMUH); the patients were aged 16+ over who attended MMUH. HbA1c, weight, complications, reason for pump usage, diabetic ketoacidosis (DKA), severe and mild hypoglycaemic episodes were investigated prior pump therapy and after therapy. HbA1c and weight were examined from start of pump therapy up until 66 months of pump therapy. Patients, who discontinued pump therapy, were excluded at times at which they discontinued from the therapy. Statistics were analysed and computed using Excel and SPSS for Windows. 315 patients were analysed.

**Results:** Analysis of the data showed a decrease in DKAs, Severe and Mild Hypoglycaemic episodes on pump therapy while HbA1c showed improvement with little weight change. Suboptimal patients were primarily selected for this therapy. Background retinopathy was the major complication found in the pre and post pump therapy group.

**Discussion:** This treatment provides benefit for T1DM patients with improvement in HbA1c, little weight gain and reduction in life-threatening diabetic events underscoring this effect.

## P31 Examining Effects of Feedback from Inhaler Compliance Assessment Device (INCA) in Asthma and COPD

### Mashari Alghuroba^1^, Isabelle Killane^2^, Richard Costello^2^

#### ^1^School of Medicine, Royal College of Surgeons in Ireland, Dublin, Ireland; ^2^RCSI Education & Research Centre, Beaumont Hospital, Beaumont, Ireland

##### **Correspondence:** Mashari Alghuroba

**Introduction:** Inhaler adherence can be described by two components: medication frequency and inhaler technique, both can be measured using the INCA device, an acoustic recording attached to Diskus inhaler that provides objective longitudinal data on an individual’s adherence. This study examined the effect of feedback on adherence in the INCA Pharmacy study, a randomised control single blinded clinical trial (n = 123).

**Methods:** Three groups were followed over six months: Demonstration group (n = 46) received educational sessions on how to use inhalers, Feedback group (n = 58) received feedback from the INCA device and educational sessions, Control group (n = 19) received usual care.

**Results:** The feedback group had the highest actual adherence rates at the end of the trial (66.3%). However, the Demonstration group had the highest increase in actual adherence from Visit 1 to 4 (7.6%).

**Discussion:** This may be explained by the Hawthorne and a ceiling effects, as the Feedback group had already high adherence rates before any interventions took place (69.5%), thus less potential to increase in adherence.

## P32 Difficult airway devices (DAD)study:A prospective convenience sample study of the use of videos in imparting psychomotor skills in airway management

### Saleh Alhumaid^1^, Peadar Gilligan^2^, Saeed Alzaabi^1^

#### ^1^School of Medicine, Royal College of Surgeons in Ireland, Dublin, Ireland; ^2^Emergency Department, Beaumont Hospital, Dublin, Ireland

##### **Correspondence:** Saleh Alhumaid

**Introduction:** Educational videos have been used as a method of teaching in many schools and universities. Usually, educational videos are based on theoretical material such as science and math. Studies prove that educational videos are effective for teaching and increasing students’ belief in their abilities. However, there is little research on the usefulness of videos in teaching skills. This study is held to assess the effectiveness of videos in teaching psychomotor skills in airway management.

**Methods:** Videos teaching airway management was edited for the study and shown to 20 volunteers with different levels on airway management. A questionnaire and a task performance were conducted after watching the videos by participants to establish the efficacy of the videos.

**Results:** 81% of attempts performed by the participants were successful after watching the videos.

**Discussion:** This shows that video educational can actually be used for teaching skills. This research is important as it encourages further studies to be conducted in this promising field.

## P33 Community-based Clerkship in Otolaryngology-Head and Neck Surgery: An analysis of medical student performance and satisfaction

### Nikita Bansal^1^, Jason Xu^2^, Paolo Campisi^3^, Ian Witterick^4^, Allan Vescan^4^, Albino Chiodo^5^

#### ^1^School of Medicine, Royal College of Surgeons in Ireland, Dublin, Ireland; ^2^University of Toronto, Toronto, Canada; ^3^Otolaryngology-Head and Neck Surgery, The Hospital for Sick Children, University of Toronto, Toronto, Canada; ^4^Otolaryngology-Head and Neck Surgery, Mount Sinai Hospital, University of Toronto, Toronto, Canada; ^5^Otolaryngology-Head and Neck Surgery, Michael Garron Hospital, University of Toronto, Toronto, Canada

##### **Correspondence:** Nikita Bansal

**Introduction:** The otolaryngology-head and neck surgery clerkship at the University of Toronto is a one-week block during the third year that can be completed at a community or tertiary site. To ensure that students at a community site received an equivalent level of education to those at a tertiary site, the performance and satisfaction of students at both sites were compared.

**Methods:** We used data from otolaryngology-head and neck surgery clerkship evaluations and grades data during the 2013/2014, 2014/2015, and 2015/2016 academic years to compare students in community hospitals to those in tertiary hospitals. The students’ grades included a clinical and written examination mark, and student evaluations. Chi-squared tests were used to compare the evaluations and the grades were compared using t-tests. A word cloud was generated from worditout.com, to illustrate the comments that students left behind in evaluations.

**Results:** According to student evaluations, a significantly larger percentage of students in community clerkships were satisfied with their rotation compared to those in tertiary sites. Students’ comments reflected frustration with the disorganization at tertiary sites. There were no significant differences found between the grades of students in either group.

**Discussion:** Students at a community site received an equivalent, if not greater, level of education to those at a tertiary site, which may be due to more clinical exposure at community sites. This indicates the potential for high quality education in community hospitals for medical students and residents, and could lead to restructuring of teaching programs in otolaryngology-head and neck surgery, as well as other specialties.

## P34 Precision medicine in inflammatory arthritis

### Ruth Carey^1^, Douglas Veale^2^

#### ^1^School of Medicine, Royal College of Surgeons in Ireland, Dublin, Ireland; ^2^Rheumatology, St Vincent’s University Hospital, UCD, Dublin, Ireland

##### **Correspondence:** Ruth Carey

**Introduction:** Rheumatoid arthritis (RA) is reported in around 40 per 100,000 and Psoriatic arthritis (PsA) in 6 per 100,000. The long term sequalae can be significant if treated sub-optimally. Patients that maintain remission are less likely to experience significant morbidity. Treatment of the inflammatory arthritides has made remarkable advances in the past 15 years with the arrival of biologic therapies making remission an achievable goal. To evaluate disease, the DAS28 (Disease activity score) was developed and evaluated by the European League Against Rheumatism (EULAR). It provides a value between 0 and 10 to indicate the level of disease activity. Remission refers to a DAS ≤ 2.6, low disease activity is a DAS > 2.6 and ≤ 3.2; moderate activity is a DAS >3.2 and ≤ 5.1; high disease activity is >5.1. The aim of the study was to assess the outcomes of patients that commenced biologics after undergoing knee arthroscopy using the DAS28.

**Methods:** Data collected at baseline included age, gender, disease duration, current therapies, CRP and ESR. Follow up was at 3, 6 and 12 months after commencement of biologic DMARD. Response was scored using the EULAR criteria, and the overall response documented in the clinical notes.

**Results:** Response rate at 3 months was 61%; 56% at 6 months and 72% at 12 months. EULAR good response was achieved in 35% of patients at 3 months, 56.3% at 6 months and 65% at 12 months.

**Discussion:** EULAR Low to moderate disease activity was achieved in over half of patients started on biologic therapies after arthroscopy. Maintaining tight control is a key strategy to prevent long term radiographic progression.

## P35 Characterisation of the P2X7 receptor, a potential anti-epileptic drug target, in a transgenic mouse model

### Claudia Condon, Marianna Alves, Yasmina Hernandez, Tobias Engel

#### Department of Physiology and Medical Physics, Royal College of Surgeons in Ireland, Dublin, Ireland

##### **Correspondence:** Claudia Condon

**Introduction:** The P2X7 receptor is an ionotrophic ATP receptor, that is involved in action potential propagation in epilepsy. It has been demonstrated that this receptor is involved in microglia activation, leading to pathological neuroinflammation. Its presence on other cell types has been demonstrated, but its presence on neurones is yet to be determined.

Manipulating the expression of the receptor will amplify its action, enabling further analysis. Increasing the receptor numbers, could potentially confirm whether it is present on neurones.

**Methods:** Using a novel transgenic mouse which over express the P2X7R gene, coupled with GFP, we compared the effect of a previously described P2X7R targeting miroRNA (microRNA-22) on the cell-specific expression of the P2X7R..Using immunohistochemical staining and confocal microscopy, we viewed the receptor expression on neurones in the hippocampus. Furthermore, we quantified microglia in the hippocampus, as well as cell death, using Iba1 and FluroJade B staining respectively.

**Results:** Brains from antagomir-22-treated mice were effectively saturated in the receptor, and it appeared to be present across all cell types, including neurones. Quantification of the FluroJade B stained cells showed cell death increased throughout the antagomir samples, whereas microglia activation was inconsistent.

**Discussion:** The images produced provided an in-depth characterisation of the P2X7 receptor under the new mouse model. Having confirmed the receptor can be present on neurones, it may be targeted to suppress the hyperexcitibility of neurones that causes epileptic seizures. This will help explore the potential of the receptor as a therapeutic target.

## P36 Relationship between Exercise Capacity, Pulmonary Function and Demographics in Adults with Cystic Fibrosis: a cross-sectional study

### Nadine Copty^1^, Lydia Cullen^2^, Ryan Fagan^2^, Elanor Styles^2^, Ronan Conroy^3^, Marie Guidon^4^

#### ^1^School of Medicine, Royal College of Surgeons in Ireland, Dublin, Ireland; ^2^Physiotherapy Department, Beaumont Hospital, Dublin, Ireland; ^3^Department of Epidemiology and Public Health Medicine, Royal College of Surgeons in Ireland, Dublin, Ireland; ^4^School of Physiotherapy, Royal College of Surgeons in Irealnd, Dublin, Ireland

##### **Correspondence:** Nadine Copty

**Introduction:** Forced expiratory volume in one second (FEV1%) has been shown to be the most important single factor predictive of survival in patients with Cystic Fibrosis. Six Minute Walk Tests (6MWTs) and Modified Shuttle Walk Test (MSWTs) play an important role in assessing and monitoring exercise capacity in patients with CF. A strong correlation exists between FEV1% and distance achieved in the MSWT in patients with moderate-to-severe lung function deficits. In this study the relationship between these exercise tests and lung function was further investigated taking into account age, gender and BMI.

**Methods:** This study is a cross-sectional study carried out in Beaumont Hospital, (Dublin, Ireland). The study included 118 6MWTs performed by 41 patients and 107 MSWTs performed by 48 patients. A Linear Regression with adjustment for clustering of measurements within patients was used to examine predictors of performance.

**Results:** Results of the 6MWT revealed that a 1% increase in FEV1% and a one-year increase in age were associated with an additional 3.7m and 3.8m respectively. As for the MSWT, a 1% increase in FEV1% was associated with an additional 10m in total distance, and males achieved a significantly greater distance than females.

**Discussion:** FEV1% and age influenced the total distance for the 6MWT while FEV1% and gender influenced the total distance for the MSWT.

## P37 Confirming Suspected Epilepsy-Related Pathogenic Variants Identified Through Next-Generation Sequencing (NGS)

### Jack Donohue^1^, Mark McCormack^2^, Norman Delanty^3^ Gianpiero Cavalleri^2^

#### ^1^School of Medicine, Royal College of Surgeons in Ireland, Dublin, Ireland; ^2^Mollecular and Cellular Therapeutics, Royal College of Surgeons in Ireland, Dublin, Ireland; ^3^Clinical Neurological Sciences, Beaumont Hospital, Dublin, Ireland

##### **Correspondence:** Jack Donohue

**Introduction:** 50 million people worldwide have epilepsy, and the majority of cases are thought to be multifactorial. By identifying genes related to epilepsy through next-generation sequencing [NGS], we can elucidate mechanisms of the disease, and better diagnose and treat patients individually. However, pathological variants recognised by NGS can occasionally be due to sequencing artifacts arising due to ineffective quality control. As a result, secondary confirmation via Sanger sequencing is an important and necessary step in verifying the legitimacy of such mutations. In this project we focused primarily on confirming small-scale mutations such as SNPs and indels.

**Methods:** DNA samples from patients with epilepsy were filtered using a bioinformatics pipeline to remove innocuous mutations from their variant libraries, thus suggesting candidate pathogenic regions. This increased the likelihood of isolating the true causative mutation(s) and involved comparing the DNA to a reference genome. Primers were then developed for regions of interest, with PCR and gel electrophoresis runs allowing us to amplify these sequences. We used Sanger sequencing to map the amplified regions after which we could determine the presence of mutations manually.

**Results:** We assessed 20 samples, and confirmed mutations in 16. Unsuccessful analyses were due to 3 events of poor sequencing quality, and 1 sequencing artifact [the latter seen in a region commonly associated with artifacts].

**Discussion:** From our high yield of positive results, it is clear that NGS is a feasible, and perhaps preferable, method of diagnosing pathogenic variants in patients suffering from genetically-driven epilepsy. This will be particularly important in promoting precision medicine.

## P38 Monitoring the Activity of Monocytes in Redox Environments by Measuring the Produced TNF-α

### Rafie Ebrahimi Ghaei^1^, Sarah O’Neill^2^

#### ^1^School of Medicine, Royal College of Surgeons in Ireland, Dublin, Ireland; ^2^Molecular and Cellular Therpeutics, Royal College of Surgeons in Ireland, Dublin, Ireland

##### **Correspondence:** Rafie Ebrahimi Ghaei

**Introduction:** The aim of this study is to see if there is a link between the TNF-α production by monocytes and the external redox environments around them. This is important in many diseases, such as Myocardial Infarction (MI), Tuberculosis (TB) or even autoimmune diseases such as Multiple sclerosis (MS) and Rheumatoid Arthritis. This investigation has shown that the redox environments do clearly play a major role in the production of TNF-α by monocytes.

**Methods:** Human monocytes were isolated from the buffy coat by using Monocyte Isolation Kit II, ordered from MACS Miltenyi Biotec. In the present paper these monocytes were exposed to different redox environments by controlled addition of Cys, CySS to the culture media and the produced TNF-α was measured using ELISA.

**Results:** Looking at the redox environments, when treated with LPS, monocytes in an oxidising Cys environment produced a higher concentration of TNF-α (242.99pg/ml) compared to a reducing Cys environment (183.74pg/ml). Results have shown that when monocytes are under oxidative stress. Using redox environments, it is possible to affect the amount of TNF-α released and therefore it is possible to control the activity of monocytes.

**Discussion:** This study used an in vitro model which relied on controlled variations in the external Cys/CySS redox state in tissue culture medium. As a result it is wise to consider the artificial nature of culture medium in contrast to blood plasma. Furthermore the response of monocytes in an oxidising or reducing Glutathione environment (using GSH/GSSG couple) is yet to be explored.

## P39 Delta-Np63 regulates hyaluronic acid synthesis by the transcriptional regulation of HAS3 in epithelial tumors

### Federica Giangrazi^1^, Mirco Compagnone^1^, Gennaro Melino^1^ Angelo Peschiaroli^2^

#### ^1^Experimental Medicine and Surgery, University of Rome “Tor Vergata”, Rome, Italy; ^2^Institute of Cell Biology and Neurobiology (IBCN), CNR, Rome, Italy

##### **Correspondence:** Federica Giangrazi

**Introduction:** p63 is a transcription factor, belonging to p53 family and it is expressed as two isoforms generated by the use of two alternative promoters: TAp63, which is able to induce apoptosis and protect oocytes from genotoxic damage, and Delta-Np63, which positively regulates cell proliferation, stemness and survival through the transcriptional control of several target genes. To find other Delta-Np63 target involved in tumorigenesis, were collected data from RNA-seq and Squamous Cell Carcinomas (SCC) data-sets analysis, which identified a correlation between Delta-Np63 and the Hyaluronic Acid Synthase type 3 (HAS3) expression.

**Methods:** The role of Delta-Np63 in HAS3 transcriptional regulation was assessed trough several molecular biology analysis, including qRT-PCR and immunofluorescence assay after Delta-Np63 silencing, identification of the responsive element (RE) for Delta-Np63 binding in human HAS3 promoter with ChIP and Luciferase assay.

**Results:** Accordingly, we found that silencing of Delta-Np63 decreases the expression of HAS3 in several SCC cell lines. Furthermore, Delta-Np63 depletion decreased the extracellular levels of Hyaluronic Acid (HA), suggesting that Delta-Np63 is able to control HA production through HAS3 transcriptional regulation. Mechanistically, by ChIP-seq and ChIP assay, we have identified a p63 RE located at 4 kbp upstream the transcription starting site in the human HAS3 promoter. This sequence was subsequently characterized by luciferase assay, confirming the transcriptional activation of HAS3 promoter by Delta-Np63.

**Discussion:** Notably, high expression of TP63 and HAS3 is a negative prognostic factor of Breast Cancers and Head and Neck SCC patients, suggesting that Delta-Np63/HAS3 axis might be functionally important to regulate epithelial tumor progression.

## P40 Evaluating the ageing effect in the osteogenic potential of mesenchymal stromal cells in response to biochemical and biophysical cues

### Deirdre Harford, Arlyng Gonzalez, Fergal O’Brien

#### Anatomy Department, Royal College of Surgeons in Ireland, Dublin, Ireland

##### **Correspondence:** Deirdre Harford

**Introduction:** The deterioration of bone due to ageing has been associated with a decline in the capacity of mesenchymal stromal cells (MSCs) to form new bone. In order to understand how ageing impacts on bone formation, we aim to evaluate the combinatorial effect of substrate rigidity (biophysical cues) and extracellular calcium (biochemical cues) in the osteogenic potential of MSCs from donors of different age.

**Methods:** MSCs from 20-30y adult (A-MSCs) and 11-12y child (C-MSCs) donors were subjected to variations in calcium concentrations (0-20 mM) and substrate rigidity (soft and stiff). In these conditions, cellular proliferation was assessed by Alamar Blue and the osteogenic potential of MSCs was evaluated by measuring alkaline phosphatase (ALP) activity.

**Results:** Results demonstrated a calcium-induced proliferation in A-MSCs and C-MSCs, with A-MSCs showing a higher proliferative rate than C-MSCs. Furthermore, cells cultured on stiff substrates and treated with 10 mM calcium demonstrated the highest ALP activity.

**Discussion:** This study demonstrated the lower proliferative ratio of C-MSCs when treated with calcium when compared with A-MSCs, suggesting the activation of differentiation process in C-MSCs. The highest ALP activity was observed when MSCs were cultured with 10 mM calcium on stiff substrates demonstrating the beneficial effect of combining calcium and substrate rigidity to enhance the osteogenic commitment of A-MSCs. Further assays are needed to investigate the effects of calcium and substrate rigidity on C-MSCs. In conclusion this project highlighted the relevance of studying the combinatorial effect of biochemical and biophysical cues to control the stem cell fate.

## P41 Standardization and optimization of thyroid nodules and cancer treatment: a chart review for quality improvement project in Michael Garron Hospital

### Yee Soo Kim^1^, Rebecca Fine^2^, Raymond Fung^2^

#### ^1^School of Medicine, Royal College of Surgeons in Ireland, Dublin, Ireland; ^2^Department of Endocrinology, Michael Garron Hospital, Toronto, Canada

##### **Correspondence:** Yee Soo Kim

**Introduction:** The American Thyroid Association (ATA) issued the newest guideline on the care of thyroid nodules and cancer in late 2015. However, no previous studies are done on practice variations within the Michael Garron Hospital. This project aims to standardize and improve the level of care for the patients with thyroid nodules and cancer, closer to the 2015 ATA guideline.

**Methods:** Retrospective chart review was performed on patients diagnosed with thyroid cancer or nodules. The following four areas were reviewed: quality of ultrasound report, adequate Radioactive Iodine (RAI) dosage, the adequate level of post-OP TSH suppression, and the malignancy rate of FNA cytology sample diagnosed as Atypia of Unknown Significant/Follicular Lesion of Unknown Significance (AUS/FLUS). Adherence to the guideline was evaluated in search of areas for improvement and possible changes to increase the quality of care.

**Results:** The ultrasound report contains on average 5.18/11 features recommended from the guideline. 12/15 patients were given the adequate RAI dosage corresponding to their risk category. Patients meeting the target TSH range at 1 year post-OP, 2 year post-OP and after the release of the newest guideline (2016) was 24%, 22% and 54.3% respectively. Malignancy rate for the AUS/FLUS sample is between 32-42%.

**Discussion:** RAI dosage, TSH suppression and the malignancy rate for AUS/FLUS are either well in-progress or already adherent to the 2015 ATA guideline. Standardization of the ultrasound report will be the key area of focus, which could be improved with a simple implementation of a template in the future steps of this study.

## P42 Modeling Chronic Inflammation: Analysis of THP-1 Macrophage Immune Markers Under Exposure to Extracellular Iron and Haem

### Cong (Johnny) Luo, Patrick Walter

#### Biology, University of Victoria, Victoria, Canada

##### **Correspondence:** Cong (Johnny) Luo

**Introduction:** Beta-thalassemia is caused by beta-globin gene mutations. These patients require frequent blood transfusions that leave them vulnerable to iron overload in various organs of the body. We hypothesised that haem and iron exposures to macrophages increase the expression of inflammatory markers and mediate chronic inflammation in patients with iron overload.

**Methods:** Macrophages from a monocytic cell line (THP-1) were exposed for 24 hours to increasing concentration of extracellular ferric citrate and haem. The exposure was done in the presence and absence of a Toll Like Receptor-4 (TLR-4) inhibitor named CLI-095. Iron loading was measured using the ferrozine assay and flow cytometry was done to determine the production of reactive oxygen species (ROS), interleukin-6 (IL-6), and TLR-4.

**Results:** Increased iron loading was seen with increased ferric citrate exposure only. An increase in ROS+ cells was seen with increased haem regardless of exposure to CLI-095. Percentage of TLR-4 expression was unchanged in both treatment cases and unaffected by CLI-095. IL-6 production in cells exposed to iron, increased in the regardless of CLI-095 exposure; however, under exposure to haem, IL-6 production increased only in the absence of CLI-095.

**Discussion:** Increased exposure to extracellular iron caused iron loading and increase in oxidative stress on THP-1 macrophages. The production of IL-6 was affected by TLR-4 downstream signalling, as seen with CLI-095 reducing IL-6 production under increased haem exposure. These results suggest that excess extracellular iron mediates inflammation through multiple mechanisms, and that haem mediates inflammation through TLR-4 signalling in THP-1 macrophages.

## P43 A dose-response study on the effects of increased soluble fiber content in cereal on laxation

### Simraaj K. Powar^1^, Peter Spadafora^2^, Robin Owen^2^, Ines Banjari^2^, Vladimir Vuksan^3^

#### ^1^School of Medicine, Royal College of Surgeons in Ireland, Dublin, Ireland; ^2^Risk Factor Modification Center, St. Michael’s Hospital, Toronto, Canada; ^3^Nutritional Sciences, University of Toronto, Toronto, Canada

##### **Correspondence:** Simraaj K. Powar

**Introduction:** The Summary of Health Statistics for U.S. Adults states the prevalence of constipation (30-40%) is higher than diabetes (8%) and coronary heart disease (6%), both of which attract more research. As a soluble fiber, psyllium can be used to reduce constipation and our study is the first to test the dose-response effect of psyllium on laxation.

Objective: A dose-response test was conducted in psyllium-enriched wheat-based cereal(PEC) on stool weight, oral-anal transit time, breath gases, and tolerability in humans. The purpose of supplementation was to enrich the North American diet though modified cereal.

**Methods:** The study followed a five-phase randomized-crossover design in 25 healthy volunteers (sex:11M:14F, age:28.1 ± 2.1years, weight:69.7 ± 2.6kg, height:170.0 ± 2.0cm, BMI:24.0 ± 0.5kg/m^2^). Participants received PEC at increasing doses (30, 60, or 90 grams with supplemental low-fiber cornflakes) or low-fiber cornflakes (90g) for breakfast. Each phase was fifteen days in duration, followed by a thirteen-day washout period. Stool collection, transit time by stool dye, diet and symptom diaries, and breath gases were collected and analyzed for each phase.

**Results:** As psyllium content increased, there was an increase in stool weight from cornflakes to PEC 90g (P < 0.05, P = 0.0079). There was a decrease in oral-anal transit time as psyllium content increased from cornflakes (30.6 ± 3.2 hours) to PEC90g (20.7 ± 3.1 hours).There were no significant changes in breath gases as psyllium content increased; although, a perceived increase in flatulence. All other symptoms were comparable between cereals.

**Discussion:** Conclusion: The 90-gram psyllium-enriched cereal portion maximally increased stool weight and decreased transit time, with low breath gases and few side effects, thus reducing constipation.

## P44 Colorectal cancer increases the thrombogenicity of platelets

### Louis Richter^1^, Jonathan Cowman^2^, Eimear Dunne^2^ Dermot Kenny^2^

#### ^1^School of Medicine, Royal College of Surgeons in Ireland, Dublin, Ireland; ^2^Molecular and Cellular Therapeutics, Royal College of Surgeons in Ireland, Dublin, Ireland

##### **Correspondence:** Louis Richter

**Introduction:** Cancer associated thromboembolism is the second highest cause of death in oncology patients, behind metastasis. It is known that patients with metastatic disease display global platelet hyperreactivity. However any previous assays used to validate this claim were non-physiological.

**Methods:** Using the novel physiological assay that measures Dynamic Platelet Function (DPFA), we compared platelet activity in a cohort of patients with metastatic colorectal cancer (n = 23) against a cohort of healthy controls (n = 21). Blood samples were incubated with a lipophilic fluorescent dye (DiOC_6_) and perfused through a von Willebrand Factor (vWF) coated flow chamber at arterial shear (1,500s^-1^). Platelet behaviour is captured via real time digital-microscopy. Only platelets that interact with the vWF coated surface are captured by the imaging software. The image is then processed by MATLAB Image Processing Toolkit.

**Results:** Platelets from the patient cohort adhered more stably to the vWF surface (p = 0.02) and travelled significantly less prior to arresting on the vWF surface (p ≤ 0.0005). Coupled with a reduced velocity of movement and a higher general cumulative platelet adherence to the surface (p ≤ 0.04 and p ≤ 0.02 respectively), it is clear that platelets from the patient cohort were more thrombogenic than controls.

**Discussion:** It has also been shown that platelet aggregation around circulating tumour cells protect against attack by cytotoxic natural killer cells, hence aiding metastasis. With potential mortality lowering benefits from cancer associated thromboembolic deaths, reduction in incidence of colorectal cancer development and reducing the tumor’s ability to metastasise; it is perhaps time to consider aspirin as part of the treatment regiment of colorectal cancer.

## P45 An audit of chest computed tomography scans in the emergency department over a 1 year interval

### Sri Vatturi^1^, Jason Seidel^2^, Mary Bissell^3^, Kathy McKay^3^, Angus Hartery^3^

#### ^1^School of Medicine, Royal College of Surgeons in Ireland, Dublin, Ireland; ^2^Memorial University of Newfoundland, St. John’s, Canada; ^3^Department of Radiology, St. Clare’s Hospital, St. John’s, Canada

##### **Correspondence:** Sri Vatturi

**Introduction:** There has been significant increase in the amount of diagnostic imaging requests from the emergency department causing a rapid increase in workload for the radiology trainee on call. This challenge presents an opportunity to analyze the requests and final results to guide radiology residency curriculum. The purpose of this study is to characterize the number of chest CT scans requested by the emergency department, assess the indications, and final diagnoses associated with these scans. These results can then be used to provide a structured baseline for the emergency radiology curriculum and training of junior residents.

**Methods:** A retrospective review was conducted on picture archiving and communications system (PACS). Data was collected on patients who presented to the ER department and who underwent a chest CT scan at 3 major hospitals in 2013. The data collected included the type of scan, indication for the scan and the final diagnosis.

**Results:** In total there were 471 scans completed. Of these, 138 were standard chest CTs, 253 were pulmonary angiograms and 31 were thoracic aortic angiograms. The most common indication was chest pain and shortness of breath (334), followed by trauma (73) followed by abdominal pain (33). The most common diagnosis was Nil acute (203) followed by malignancy (51) followed by pulmonary embolus (34).

**Discussion:** Analyzing the indications and final CT diagnosis of emergency radiology computed tomography can reveal the most commonly suspected, and final CT diagnoses. This in turn can be used to optimize training and call preparation for junior radiology residents.

## P46 Investigating the Role of Matrix Metalloproteinases in Mammary Microcalcification

### Ahmad AlAbdulkareem^1^, Shane O’Grady^2^, Maria Morgan^2^

#### ^1^School of Medicine, Royal College of Surgeons in Ireland, Dublin, Ireland; ^2^Department of Molecular and Cellular Therapeutics (MCT), Royal College of Surgeons in Ireland, Dublin, Ireland

##### **Correspondence:** Ahmad AlAbdulkareem

**Introduction:** Despite the strong association of microcalcifications with breast cancer, to date investigations of the genesis and nature of those calcifications have been very limited. Gelatinases (also known as matrix metalloproteinase-2&9) have been previously shown to influence pathological calcification in developing atherosclerotic plaques and vascular smooth muscle cells. This current study aims to investigate the relationship between gelatinases and mineralizations by mammary adenocarcinoma cell line MDA-MB-231 in vitro.

**Methods:** An osteogenic cocktail of beta-glycerophosphate (beta-GP) and ascorbic acid supplemented with dexamethasone was used to induce calcification with or without gelatinases inhibitor SB-3CT. To assess calcification, cells were routinely stained with Alizarin red and von Kossa stains. Calcium content, Alkaline Phosphatase (ALP) activity levels and MMP2 expression levels were also quantified.

**Results:** Contrary to previous work done on calcifying vascular smooth muscle cells, gelatinases inhibition with SB-3CT promoted calcification in mammary adenocarcinoma cells yet it inhibited expression of MMP2 mRNA.

**Discussion:** Experimenting with different concentrations or with other gelatinases inhibitors might be necessary to better understand the complexity of the relationship between gelatinases and microcalcifications. It may also be possible that gelatinases do not exhibit the same level of effect on calcifications in mammary adenocarcinoma cell as they do in vascular smooth muscle cells or that they possess an opposite effect, however, more verification is needed.

## P47 A review of Massive Obstetric Haemorrhage (MOH) in the East of Ireland and its association with Maternal Obesity

### Sarah Alnafisee^1^, Cathy Monteith^1^, Elizabeth Tully^1^, Colin Kirkham^2^, Fergal Malone^1^

#### ^1^Department of Obstetrics & Gynaecology, The Rotunda Hospital, Royal College of Surgeons in Ireland, Dublin, Ireland; ^2^Department of Research, The Rotunda Hospital, Dublin, Ireland

##### **Correspondence:** Sarah Alnafisee

**Introduction:** Massive obstetric haemorrhage (MOH), blood loss of >2000ml, is a life-threatening emergency in the postpartum. The aim of this review is to address the incidence of maternal obesity, a modifiable risk factor contributing to MOH.

**Methods:** This 6-year retrospective review involved the interrogation of the annual clinical reports of the tertiary maternal centres in the East of Ireland between the years 2009-2014. We assessed patient risk factors for developing MOH in the antenatal period with a focus on maternal obesity (Body Mass Index (BMI) ≥30 Kg/m^2^). Associations between categorical variables were tested using Pearson’s chi-square test.

**Results:** The incidence of MOH was 2.21/1,000 livebirths during the 6-year period. Of those women 20.5% of cases had BMIs recorded and 34.72% of those with recorded BMI were obese. Within the obese cohort, patients suffered an average blood loss of 2820ml in the first 24 hours postpartum, with 88% requiring a blood transfusion. There was a significant association between maternal obesity and developing MOH: (X^2^ (1) = 32.63, p-value < 0.001).

**Discussion:** Maternal obesity is a preventable risk factor that contributes to MOH. As detailed in the most recent report by the World Health Organization (WHO) presented at the 2015 European Congress on Obesity, there’s a predicted rise in obesity for women in Ireland from 23% to 57% by the year 2030. Pre-conception modification strategies for maternal obesity could potentially decrease the incidence of MOH and improve obstetric outcomes.

## P48 The relationship between Cognitive Function and QoL in patients with pituitary tumours undergoing transsphenoidal surgery; a prospective study

### Richard Bresler^1^, Michael Cusimano^2^

#### ^1^School of Medicine, Royal College of Surgeons in Ireland, Dublin, Ireland; ^2^Neurosurgery Department, University of Toronto, Toronto, Ontario

##### **Correspondence:** Richard Bresler

**Introduction:** The relationship between cognition, quality of life and endoscopic transsphenoidal resection of tumors of the pituitary gland remains unclear. The authors assessed the impact of cognition on quality of life, using the CFQ and SF-36 questionnaire respectively, and the effect of endoscopic transsphenoidal surgery on these two health-outcomes measures.

**Methods:** This single-centre prospective study included 61 patients who underwent their first endoscopic transsphenoidal surgery between January 2010-March 2016. Patients completed the CFQ and the SF36 questionnaires pre-operatively and post-operatively at a mean of 117.3 days (SD = 138.48) during their first clinical follow-up in the Neurosurgical Pituitary Clinic.

**Results:** 27 males (44.3%) and 34 females (55.7%) underwent endoscopic transsphenoidal surgery at a mean age of 47.87 years (SD = 14.45).CFQ scores significantly improved following endoscopic transsphenoidal resection (p = .0104). Endoscopic transsphenoidal surgery did not significantly improve total SF-36 scores (p = .3527). However, significant improvements were found in the mental health (p = .014) and general health (p = .0071) subdomains. A strong negative correlation was found between pre-operative CFQ score and total SF36 score (r = -0.65228, p = <.0001), and post-operative CFQ score and total SF36 score (r = -0.6314,p = <.0001).

**Discussion:** Endoscopic transsphenoidal approach significantly improves cognitive function and the mental health and general health domains of SF-36 health measures. Moreover, impaired cognitive function negatively impacts quality of life. The relationship between cognition and quality of life and the effectiveness of endoscopic transsphenoidal surgery on health-outcome measures needs to be examined further.

## P49 Hormonal Therapy Medication Taking Behavior and Health Related Quality of Life In Women With Stage I to III Breast Cancer In Ireland

### Ang Bee Hong^1^, Kathleen Bennett^1^, Stephan U Dombrowski^2^, Linda Sharp^3^, Caitriona Cahir^1^

#### ^1^Division of Population Health Sciences, Royal College of Surgeons in Ireland, Dublin, Ireland; ^2^Division of Psychology, University of Stirling, Stirling, Scotland; ^3^Institute of health and society, Newcastle University, Newcastle upon Tyne, UK

##### **Correspondence:** Ang Bee Hong

**Introduction:** Taking adjuvant hormonal therapy for 5-10 years is recommended to prevent breast cancer recurrence in those with estrogen positive disease. However, despite the proven clinical efficacy of adjuvant hormonal therapy many women do not take their treatment as recommended. The aim of this study was to investigate the association between adjuvant hormonal therapy medication taking behavior (MTB) and health related quality of life (HRQOL) in women with stage I-III breast cancer.

**Methods:** A cross-sectional national population-based study of women with stage I-III breast cancer prescribed adjuvant hormonal therapy in Ireland. Participants were identified from the National Cancer Registry Ireland database and invited to complete a postal questionnaire measuring; (i) demographics; (ii) self-reported MTB; and (iii) HRQOL using the Functional Assessment of Cancer Therapy-Breast (FACT-G). Ethical approval was granted by the Irish Congress of General Practitioners. The association between MTB and HRQOL was assessed using linear regression analysis with adjustment for demographic and clinical covariates.

**Results:** In total 1,612 women completed the questionnaire (response rate = 65%); 1,207 (74.9%) women were adherent and persistent; 178 (11%) women were non-adherent but persistent and; 227 (14.1%) women were non-persistent. Women who were non-adherent had a statistically significant lower quality of life (co-efficient -2.06, SE 0.69, p < 0.01) compared to women who were adherent after adjusting for covariates.

**Discussion:** For oncologists, women may be non-adherent to their hormonal therapy due to the perceived adverse effects of their therapy and reduced HRQOL. Interventions need to be developed to help women take their treatment.

## P50 Epidemiological and Clinical Features of Medium Chain Acyl-CoA Dehydrogenase Deficiency in the Paediatric Population of the Republic of Ireland

### Zhubene Mesbah^1^, Kam Sing-Ho^1^, Patricia Fitzsimons^2^, Philip Mayne^3^, Ellen Crushell^4^

#### ^1^School of Medicine, Royal College of Surgeons in Ireland, Dublin, Ireland; ^2^Temple Street Children’s University Hospital, Dublin, Ireland; ^3^Temple Street Children’s University Hospital, Royal College of Surgeons in Ireland, Dublin, Ireland; ^4^National Centre for Inherited Metabolic Disorders, Temple Street Children’s University Hospital, University College Dublin School of Medicine, Dublin, Ireland

##### **Correspondence:** Zhubene Mesbah

**Introduction:** Medium Chain Acyl-CoA Dehydrogenase Deficiency (MCADD) is a recessive disorder of fatty acid oxidation with a significant risk of mortality at first clinical presentation. This is of particular concern in countries such as Ireland that do not screen newborns for MCADD. The current study aims to investigate the disease frequency of MCADD in the Irish setting and inform policy regarding the addition of MCADD into the Irish National Newborn Bloodspot Screening Program.

**Methods:** Children (<18 years) with MCADD were identified via the National Centre for Inherited Metabolic Disorders and the metabolic laboratory at Temple Street Children’s University Hospital. CSO population data was used to calculate epidemiological figures.

**Results:** From 1/1/1998 to 30/8/2016, 17 children were diagnosed with MCADD in Ireland. The average age at clinical presentation was 1.48 years (Range: 0.005 to 2.86) with 2 patients diagnosed post mortem. The incidence of MCADD during this period was 1:71650 with a mortality of 15.38% in the first clinical presentation- no child died post diagnosis. The common c.985A > G mutation accounted for 88% of alleles. The current prevalence of MCADD was calculated to be 1.23 per 100,000 children.

**Discussion:** The incidence of MCADD in Ireland is lower than global estimates (incidence of 1:10,000 - 30,000 in most countries where newborn screening is in place). The potential for under-ascertainment and late diagnosis of cases exists in Ireland and is of concern for a treatable condition with a significant risk of mortality when undiagnosed.

## P51 Force required to be applied to a novel Wireless Autoclavable Palpation device to localise lung tumours in minimally invasive surgeries

### Deena Shah^1^, Anish Naidu^2^, Abelardo Murillo^3^, Michael D Naish^3^, Rajni Patel^3^

#### ^1^School of Medicine, Royal College of Surgeons in Ireland, Dublin, Ireland; ^2^Schulich School of Medicine, London, Canada; ^3^Canadian Surgical technologies and Advanced Robotics (CSTAR), University Hospital, London, Canada

##### **Correspondence:** Deena Shah

**Introduction:** Minimally invasive surgery (MIS) is currently at the forefront as the preferred method of treatment for early-stage lung cancer. During a surgery for tumour resection, both preoperative imaging and intraoperative manual palpation are required for accurate localization of the tumor. In MIS, manual palpation is not possible, and as a result large amount of healthy lung tissue is unnecessarily removed. Tools that allow for intraoperative minimally invasive localization of both large and small lung tumours are currently being tested.

**Methods:** This study evaluates a novel, wireless, autoclavable palpation device, which provides tactile and kinesthetic feedback in visual form to aid in the localization of tumours. This device is inserted into the patient via an incision similar in size to that of a laparoscopic instrument used during MIS.

In this study, porcine lung and liver samples were utilized to test the amount of force required to localize agar-iodine phantom tumours of 5 mm, 10 mm and 15 mm diameter, each at depths of 5 mm, 10 mm and 15 mm.

**Results:** It was determined that the 5 mm tumours required higher amounts force for localization (1.3 - 4 N) at all depths, in comparison to the larger 15mm tumours (1.0 - 3.2 N). It was also determined that a force larger than 5 N resulted in permanent tissue damage.

**Discussion:** Thus, the autoclavable palpation device presented herein can successfully localize smaller tumours within force ranges that do not cause tissue damage, and would therefore be useful for tumour localization in minimally invasive surgical procedures.

## P52 Surgery at a District Level in Malawi: Findings from the COST-Africa Project

### Rachel Dharamshi, Michael Strader, Jakub Gajewski, Tracey McCauley

#### Epidemiology Department, Royal College of Surgeons in Ireland, Dublin, Ireland

##### **Correspondence:** Rachel Dharamshi

**Introduction:** Sub-Saharan Africa continues to struggle with meeting the surgical needs of its population. In response, there has been an increased demand for the training of non-physician clinicians. COST-Africa a research project sponsored by EU/FP7, seeks to provide training to non-physician clinicians -- Clinical Officers (CO) in Zambia and Malawi with the goal of increasing surgical capacity. This paper compares data from Malawi hospitals in order to characterize patient populations, common conditions, and types of procedures.

**Methods:** 17 COs underwent training in basic sciences and completed two years of in-service surgical training. Data from operating theatres were collected for 24 months, (Jan 2014-Dec15), including patient demographics, presenting conditions, and types of procedures. Data was collected from 8 district hospitals within Malawi and trends were analyzed within Excel.

**Results:** The most common conditions across all Malawi hospitals (N = 8) were prolonged labor, incomplete, and retained products of conception. The most common procedure across all hospitals was C-Section. The average age ranged from 23.73-24.91 between hospitals. Spinal anesthetics were used most for C-Sections in all hospitals except for Mchinji. Females made up the majority (85.38%) of the patient population in each hospital.

**Discussion:** The majority of patients across all hospitals were female. As such, it is not surprising that obstetric/gynecological conditions were the most common presentations at each hospital. C-sections, the “bread and butter” of district hospital surgery, was the most common procedure, consistent with current literature. Although most hospitals used spinal anesthetic for C-sections, Mchinji used general, which might be indicative of resource accessibility. Further analysis of the data and additional context could provide a better picture of current challenges faced in Malawi hospitals.

## P53 Passport or Not? Abdominal Aortic Aneurysm Surveillance

### Jonavan Tan^1^, Daragh Moneley^1^, Austin Leahy^1^, Patricia Fitzgerald^2^

#### ^1^Department of Surgery, Beaumont Hospital, Royal College of Surgeons in Ireland, Dublin, Ireland; ^2^Non Invasive Vascular Unit, Deptartment of Surgery, Beaumont Hospital, Royal College of Surgeons in Ireland, Dublin, Ireland

##### **Correspondence:** Jonavan Tan

**Introduction:** Abdominal aortic aneurysms (AAA) are a common, potentially life-threatening condition. The major risk in AAA is rupture, which increases with increasing aneurysm diameter. Regular surveillance (via abdominal ultrasound) helps appropriate timing of intervention -- generally indicated at 5.5cm due to estimated 11% annual rupture risk. A previous audit of two Vascular Surgical Clinics (VSCs) and an AAA Specific Clinic (ASC) showed poor scheduling adherence to AAA surveillance guidelines. It was concluded that in addition to clarification of surveillance intervals to teams, an AAA-specific patient passport should be designed, to further ensure improvements in scheduling adherence and patient safety.

**Methods:** A patient passport was designed, road-tested and distributed. Audit of the ASC was performed.

**Results:** Existing disease-specific passports were reviewed for sections applicable to AAA. Passports were designed and road-tested for patient feedback, incorporated into the final design. A driver diagram was designed to aid the passport distribution process, which commenced in April 2016. Passport effectiveness will be determined through review of the passport design, distribution and usage, and a re-audit of clinics.

**Discussion:** The passport may enhance awareness and promote healthier lifestyle changes to reduce AAA-related mortality, and ensure patients receive appropriate follow-up.

Review of passport design, distribution and usage, and re-audit of clinics, is scheduled for December 2016 to determine passport effectiveness. High-risk and/or VSC patients will likely see greater benefit. Additional booklets may be designed for follow-up post-EVAR. We anticipate few balancing measures; Concerns include potential record-keeping inconsistencies and patient confidentiality.

## P54 Neuorological and Neuropsychiatric Manifestations of Tuberous Sclerosis

### Nicola A. Walsh^1^, Claire Kirk^2^, David Webb^3^, Andrew Green^2,4^

#### ^1^School of Medicine, Faculty of Health Sciences, Trinity College Dublin, Dublin, Ireland; ^2^National Centre for Medical Genetics, Our Lady’s Childrens Hospital Crumlin, Dublin, Ireland; ^3^Department of Neurology, Our Lady’s Childrens Hospital Crumlin, Dublin, Ireland; ^4^UCD Conway Institute of Biomolecular and Biomedical Research, Dublin, Ireland

##### **Correspondence:** Nicola A. Walsh

**Introduction:** Tuberous sclerosis complex (TSC) is a genetic disorder affecting 1/6,000 live births caused by mutations in either TSC1 or TSC2 genes (1-3). Neurological and neuropsychiatric manifestations of TSC are associated with high morbidity and mortality (4). The aim of this study was to characterise the phenotype of patients with TSC in Ireland.

**Methods:** A medical record search was carried out for patients diagnosed with TSC in the National Centre for Medical Genetics in Ireland. Records with insufficient clinical data were excluded. Characteristics examined included, information on CNS manifestations including subependymal nodules (SEN), cortical tubers, subependymal giant cell astrocytoma (SEGA), neuropsychiatric manifestations and other comorbidities.

**Results:** The database search yielded 224 records, of which 87 records were excluded, leaving 147 eligible patient records (53% female, median age 18 years). Of these, 68% had neuropsychiatric diagnoses. Of the patients in our sample with brain imaging (n = 109), 50.4% had SEN, 63.7% had cortical tubers, and 14.3% had SEGA. No significant genotype-phenotype relationship was found when comparing TSC1 and TSC2.

**Discussion:** This study demonstrates that there is a significant burden of neurological disease associated with mutations in TSC1 and TSC2. Common and significant neurological pathology identified among our patient cohort include SEN, cortical tubers and SEGA. Neuropsychiatric conditions, including epilepsy, are prevalent in this population. This is the first study characterising the phenotype of the TSC patient population that has been carried out in Ireland and identifies specific needs in this patient population.

## P55 An audit on the use of Thromboembolic Deterrent Stockings (TEDS) in surgical patients at Beaumont Hospital

### Andrew Zayas^1^, Steven Anderson^1,2^, Arnold Hill^2^

#### ^1^School of Medicine, Royal College of Surgeons In Ireland, Dublin, Ireland; ^2^Department of Surgery, Beaumont Hospital, Royal College of Surgeons in Ireland, Dublin, Ireland

##### **Correspondence:** Andrew Zayas

**Introduction:** Venous Thromboembolism (VTE) is a major entity in healthcare settings and its complications account for a significant proportion of post-operative mortality. TEDS have been shown to reduce the incidence of life-threatening VTE, but evidence suggests their usage could be improved in most institutions. This audit aims to examine patient compliance with TEDS and doctors’ prescribing habits.

**Methods:** Over two weeks, compliance was assessed in all patients admitted under general surgery, as well as doctors’ prescribing of TEDS. As an intervention, an assessment sheet was introduced before a second audit was conducted.

**Results:** There were a total of 154 and 122 patients in the pre- and post-intervention groups, respectively. Compliance rates were 49% in both audits whereas appropriate prescribing of TEDS increased from 75% to 81%. The TEDS Assessment Sheet was found on the patient bedside only 2% of the time, of which 74% were properly filled in.

**Discussion:** There is room for improvement in all aspects of TEDS usage. The poor uptake the TEDS Assessment Sheet demonstrates that our intervention did not have the desired effect, and emphasises the need for a more robust measure to enforce correct prescribing and compliance to TEDS among healthcare staff and patients, respectively.

## P56 Triplets and Beyond

### Fiona Rawat^1^, Joan O’Brien^2^, Karen Flood^2^, Elizabeth Tully^1^

#### ^1^Royal College of Surgeons in Ireland, Dublin, Ireland; ^2^Obstetrics and Gynaecology, The Rotunda Hospital Dublin, Dublin, Ireland

##### **Correspondence:** Fiona Rawat

**Introduction:** The incidence of multiparous pregnancies has increased dramatically over the last few decades. This has been largely attributed to the use of artificial reproductive techniques (ART). These pregnancies may be associated with complications such as premature delivery, perinatal morbidity and mortality as well as maternal complications all of which has a significant impact on service delivery. The aim of this study was to evaluate the prevalence and delivery outcomes of this cohort in a tertiary Maternity hospital in Dublin.

**Methods:** A retrospective audit was performed to review all high order multiple pregnancies attending for antenatal care at the Rotunda Hospital between 2009 and 2014. This descriptive study evaluated triplet and quadruplet pregnancies, with respect to age, mode of conception, chorionicity and amnionicity, gestational age at delivery, and mode of delivery.

**Results:** A total of 52 multiparous pregnancies were managed by the Rotunda Hospital during the time period, 50 triplet and 2 quadruplet pregnancies. Information regarding the mode of conception, chorionicity and amnionicity, and the mode of delivery can be found in the table below. One pregnancy ended in miscarriage at the 12 week scan. Ten further pregnancies reduced to a twin or singleton pregnancy in the first and second trimesters. From the remaining 41 intact higher order pregnancies, 36 reached viability beyond 24 weeks gestation. Five pregnancies had incomplete data to allow inclusion in the analysis.

**Discussion:** The data obtained shows that despite an increase in the use of ART since 2009, the majority of pregnancies n = 42 (81%) were actually due to natural conception. In terms of mode of delivery, emergency (n = 16 (51%)) and elective caesarean sections (n = 15(49%)) were similar in numbers. The average gestational age of delivery for elective C-sections was 34 + 1 weeks and 30 + 5 weeks for emergency C-sections. This could be a potential area of service improvement.

