# Erratum to: P47 A review of Massive Obstetric Haemorrhage (MOH) in the East of Ireland and its association with Maternal Obesity

**DOI:** 10.1186/s12919-017-0073-x

**Published:** 2017-07-10

**Authors:** Sarah Alnafisee, Cathy Monteith, Elizabeth C. Tully, Colin Kirkham, Fergal D. Malone

**Affiliations:** 1 Department of Obstetrics & Gynaecology, The Rotunda Hospital, Royal College of Surgeons in Ireland, Dublin, Ireland; 2 Department of Research, The Rotunda Hospital, Dublin, Ireland

## Erratum

After publication of the below abstract in supplement [1], it was brought to our attention that in abstract P47, two authors have middle initials they would like included. The third and fifth authors are published as Elizabeth Tully and Fergal Malone, but should have been Elizabeth C. Tully and Fergal D. Malone.

## P47 A review of Massive Obstetric Haemorrhage (MOH) in the East of Ireland and its association with Maternal Obesity

### Sarah Alnafisee^1^, Cathy Monteith^1^, Elizabeth Tully^1^, Colin Kirkham^2^, Fergal Malone^1^

#### ^1^Department of Obstetrics & Gynaecology, The Rotunda Hospital, Royal College of Surgeons in Ireland, Dublin, Ireland; ^2^Department of Research, The Rotunda Hospital, Dublin, Ireland

##### **Correspondence:** Sarah Alnafisee

**Introduction:** Massive obstetric haemorrhage (MOH), blood loss of >2000 ml, is a life-threatening emergency in the postpartum. The aim of this review is to address the incidence of maternal obesity, a modifiable risk factor contributing to MOH.

**Methods:** This 6-year retrospective review involved the interrogation of the annual clinical reports of the tertiary maternal centres in the East of Ireland between the years 2009-2014. We assessed patient risk factors for developing MOH in the antenatal period with a focus on maternal obesity (Body Mass Index (BMI) ≥30 Kg/m2). Associations between categorical variables were tested using Pearson’s chi-square test.

**Results:** The incidence of MOH was 2.21/1,000 livebirths during the 6-year period. Of those women 20.5% of cases had BMIs recorded and 34.72% of those with recorded BMI were obese. Within the obese cohort, patients suffered an average blood loss of 2820 ml in the first 24 hours postpartum, with 88% requiring a blood transfusion. There was a significant association between maternal obesity and developing MOH: (X2 (1) = 32.63, p-value < 0.001).

**Discussion:** Maternal obesity is a preventable risk factor that contributes to MOH. As detailed in the most recent report by the World Health Organization (WHO) presented at the 2015 European Congress on Obesity, there’s a predicted rise in obesity for women in Ireland from 23% to 57% by the year 2030. Pre-conception modification strategies for maternal obesity could potentially decrease the incidence of MOH and improve obstetric outcomes.
